# Dynamic analysis and driving performance verification of multi-functional electric tracked vehicles for greenhouses in hilly and mountainous areas

**DOI:** 10.3389/fpls.2026.1805450

**Published:** 2026-04-10

**Authors:** Lingkun Zeng, Jingbin Sun, Yongqiang He, Jing Ying, Qun Sun, Shengyang Sun, Jingbo Zhou, Zengzhi Guo, Fei Mu

**Affiliations:** 1 College of Mechanical and Automotive Engineering, Liaocheng University, Liaocheng, China; 2 College of Agricultural Engineering, Shanxi Agricultural University, Taigu, China; 3 Shanxi Key Laboratory of Key Technologies and Equipment for Agricultural Machinery, Taigu, China; 4 Key Laboratory of Agricultural Equipment Technology for Hilly and Mountainious Areas, Ministry of Agriculture and Rural Affairs, Chengdu, China; 5 Sichuan Academy of Agricultural Machinery Sciences, Chengdu, China; 6 School of Computer Science and Technology, Harbin Institute of Technology, Harbin, China

**Keywords:** driving performance, dynamic analysis, electric tracked vehicles, greenhouses, hilly and mountainous areas

## Abstract

Hilly and mountainous areas account for approximately one-third of China’s total cultivated land, where crops such as tobacco, tea, fruit trees, and tall-stalk varieties such as sorghum are extensively cultivated. However, the level of mechanization in crop production remains relatively low, primarily because of the poor adaptability of existing agricultural machinery to rugged terrain. Conventional crawler chassis struggle to simultaneously meet the requirements of high ground clearance and operational stability, and their limited functionality further restricts efficient agricultural production in these regions. To address these challenges, this paper presents a novel high-clearance electric crawler chassis designed for greenhouse hilly and mountainous areas. The chassis employs a gantry frame with a ground clearance of 1550 mm, facilitating operation over tall row crops. It adopts a dual-motor independent drive scheme, ensuring the continuity of power and achieving in-place steering. A longitudinal center-of-gravity adjustment device driven by dual electric actuators allows the frame to shift ±350 mm relative to the crawler traveling system. Multibody dynamics simulations conducted in RecurDyn 2023 demonstrated that the chassis can achieve a maximum climbing angle of 38°, crossing for trenches up to 800 mm wide and can surmount vertical obstacles up to 300 mm high. Field tests carried out on slopes in accordance with Chinese National Standard GB/T 3871–2004 revealed that the longitudinal center-of-gravity adjustment device significantly enhanced the driving performance of the high-clearance crawler chassis. When activated, the maximum safe climbing angle increased from 30° to 35°, the maximum obstacle surmounting height at 3.6 km/h increased from 250 mm to 300 mm, and the maximum trench-crossing width increased from 600 mm to 700 mm. At 5.4 km/h, the trench-crossing width increased from 700 mm to 750 mm. The field test results were consistent with the simulation outcomes. The development of this high-clearance crawler chassis effectively reconciles the competing demands of high ground clearance and stability while improving overall versatility. It provides valuable technical insights for the design of safe and efficient agricultural machinery suited to complex terrains in hilly and mountainous regions, contributing significantly to the advancement of agricultural mechanization and modernization in these areas.

## 1 Introduction

The agricultural power chassis, which serves as the core driving unit in agricultural production, plays a critical role in delivering high-efficiency driving performance while supporting the attachment of diverse implements and ensuring chassis stability. These capabilities are essential for meeting the varied operational demands of different agronomic processes (Jiang et al., 2025). In China, hilly and mountainous regions account for a substantial proportion of the country’s arable land, representing approximately one-third of the total cultivated area (Sun et al., 2023). Such areas are characterized by extensive cultivation of high-stem cash crops, including tea plants, fruit trees, and oil-tea camellia (Peng et al., 2024; Zheng et al., 2020; Xiang et al., 2025). However, complex terrain and steep slopes pose significant challenges for conventional chassis, resulting in limited trafficability, poor adaptability, and low operational efficiency. In addition, the field operations in the later stages of tall crops require the chassis to have sufficient ground clearance, good inter-row passage, and reasonable working space to avoid mechanical damage to the crop stems and canopies during operations, but existing chassis are difficult to balance the agricultural requirements while maintaining the driving stability in complex terrains. These constraints seriously impede regional agricultural development toward higher efficiency and quality (Li et al., 2022). Therefore, while maintaining the dynamic performance and stability of power chassis systems, enhancing their operational adaptability for high-stem crops, achieving multifunctional operation, and improving environmental sustainability are imperative. These aspects represent the primary development direction for power chassis tailored to hilly and mountainous regions (Ding et al., 2025).

In response to the aforementioned technical requirements for agricultural power chassis, the global research community has conducted extensive studies on the design and application of such chassis, achieving significant progress.

In response to the adaptive design requirements for power chassis systems, researchers have developed specialized chassis with diverse architectural configurations. Xu et al. (2024) designed a high-clearance, four-wheel independent-drive agricultural chassis to overcome the inefficiency of large machinery and enhance adaptability for broccoli cultivation in the hilly and mountainous regions of Zhejiang. To meet operational demands such as pesticide application and fertilization in tall-stalk crops, Chen et al. (2020) developed a large high-clearance self-propelled sprayer with an “X”-type hydrostatic four-wheel drive system. Jin et al. (2022) introduced a wheel-track high-clearance windrower for rapeseed to improve adaptability to clay soils within rice–rapeseed rotation systems in the middle and lower Yangtze River Basin. He et al. (2023) proposed a four-wheel self-steering (4WSS) electric chassis to mitigate issues such as the immobilization of and damage to traditional high-clearance sprayers in muddy environments. To address the poor terrain adaptability of viticultural equipment, Li et al. (2024) developed a gantry-tracked multifunctional platform capable of spanning grape trellises. To enhance power support in king grass harvesting, Wang et al. (2023) designed a crawler-type gentle slope harvester featuring an inverted trapezoidal rubber track mechanism and an HST continuously variable transmission system.

In response to the design requirements for the trafficability performance of power chassis in complex terrain environments, researchers have carried out in-depth studies on the trafficability characteristics of related chassis systems. Mou et al. (2023) performed theoretical analysis, simulation modeling, and field experiments on a crawler chassis under slope climbing, obstacle crossing, and ditch spanning conditions in mountainous orchards, thereby validating its traversing performance. Using theoretical analysis and RecurDyn simulations, Su & Huang et al. (2024) investigated the trafficability of a miniaturized crawler chassis with terrain-adaptive capabilities across complex topographies—including longitudinal and transverse slopes, trenches, and vertical obstacles. On the basis of similarity theory, Zhang et al. (2023) developed a 1:4 scaled-down platform model and conducted systematic experiments under typical working conditions such as longitudinal slope climbing, trench crossing, and ridge traversing in cohesive soil to comprehensively evaluate its trafficability. Han et al. (2022a) designed an adjustable center-of-gravity crawler chassis for mountainous orchards, theoretically analyzed key structural parameters affecting slope traversal and obstacle negotiation, and validated its performance in slope navigation, step climbing, and trench crossing through field tests. To address issues such as inadequate driving stability in complex tea plantation environments, Wu et al. (2025) studied the trafficability of a four-track tea-picking chassis through both simulations and field tests focused on slope climbing, obstacle crossing, and trench traversing, offering methodological references for research on high-clearance chassis mobility.

To address the stability design requirements of power chassis on unstructured and complex terrains, researchers have developed a variety of chassis posture adjustment mechanisms. By designing a novel articulated chassis capable of active pitch and yaw motion as well as passive torsion, Lin et al. (2022) addressed the stability limitations of conventional forestry chassis in complex terrain. In response to the inadequate stability of traditional chassis in the ridge-farming environments of southern mountainous areas, Li et al. (2024) developed an autonomous posture-adjustable wheeled chassis based on a dual-parallelogram mechanism, a dual-leadscrew system, and independent leg columns. Paul & Machavaram et al. (2024) solved the sinking and overturning problems faced by wheeled combine harvesters under muddy conditions by designing a posture-adjustable crawler chassis that uses four hydraulic cylinders to regulate the height and lateral tilt angle. Xie et al. (2023) addressed the challenges posed by complex terrain and small plot sizes that limit the operation of large and medium-sized agricultural machinery in hilly areas. They developed an arched-waist power chassis that combines crawler and wheel systems to significantly improving its terrain negotiation capability. To enhance the environmental adaptability of conventional agricultural chassis, Pan et al. (2024) designed a novel bionic wheel-legged chassis inspired by the jumping mechanism of grasshopper hind legs and incorporated a pneumo-hydraulic system. The experimental results validated its superior adaptability in unstructured environments. Sun et al. (2020; 2021) designed a vehicle body attitude adjustment device based on a parallel four-bar mechanism to address the issues of difficult leveling and poor stability of crawler tractors in hilly and mountainous areas. The structural reliability of the device was verified through a combination of theoretical mechanics analysis, finite element simulation, and orthogonal tests on a physical model. Subsequently, a remotely - controlled omnidirectional leveling mountain crawler tractor based on the parallel four - bar and double - frame mechanisms was successfully developed. Tests indicate that the tractor can effectively level the vehicle body on both transverse and longitudinal slopes, significantly enhancing the stability and operational convenience during slope operations.

In response to the design requirements for power chassis universality, researchers have developed a range of modular attachment devices and interchangeable mechanisms. Gao et al. (2020) designed a fully tracked modular agricultural power chassis based on the transmission system of traditional articulated mountain tractors. Equipped with a front quick-connect device and a PTO three-point hitch, it enables the interchange of various implements and integrates operations such as land preparation and weeding, thereby meeting diverse operational demands in hilly and mountainous areas. Roul and Singh (2022) developed a self-propelled hydraulic multi-functional chassis with an adjustable wheelbase and high ground clearance to address low machinery utilization in operations including tilling, weeding, and spraying for tall-stem crops in India. This chassis is suitable for field crops up to 2 meters in height and supports operations such as spraying, weeding, and harvesting. It also features adjustable ground clearance and track width to accommodate varying planting spacings and heights of horticultural crops. Cui et al. (2019) developed a multi-functional electric platform for greenhouse operations to mitigate issues such as high labor intensity and low operational efficiency in the management, harvesting, and transportation of greenhouse produce. The platform performs tasks including elevated picking and traction transport, achieving multi-purpose functionality with a single machine. Mengoli et al. (2020) designed a tracked orchard robot incorporating a shared power transmission system and a universal mechanical interface to enable rapid attachment changes for operations such as branch crushing and pesticide spraying. Calciolari et al. (2024) developed a modular autonomous mobile robot capable of replacing implements and adjusting their positions within a consistent structural framework. This robot performs various tasks in vineyards, including pruning and spraying, thereby supporting multi-purpose operation with a single platform.

In conclusion, the agricultural power chassis currently under research and application can be broadly classified into three types: wheeled, tracked, and wheel–track composite. While these chassis configurations have been widely deployed in field environments, they continue to face significant challenges when they are operated in the complex terrain of hilly and mountainous areas. Wheeled chassis often suffer from inadequate power performance, wheel slippage, and sinking because of insufficient adhesion. Their high ground pressure also leads to severe soil compaction, further limiting their adaptability to the varied working conditions typical of hilly and mountainous regions. A conventional crawler chassis offers a larger ground contact area and lower ground pressure, resulting in superior trafficability on soft and uneven surfaces and enhanced obstacle-crossing capability on rugged slopes. However, their generally low center of gravity and limited ground clearance hinder their suitability for interrow operations during the mid-to-late growth stages of tall-stem crops. Furthermore, most existing tracked power chassis developed to date lack posture or center-of-gravity adjustment mechanisms, restricting their ability to actively adapt to terrain variations. This limitation compromises operational stability and safety in uneven landscapes. Additionally, the majority of available power chassis are designed for singular functions, making it difficult to meet the diverse agronomic requirements associated with cash crop cultivation in hilly and mountainous areas.

In response to the aforementioned challenges, particularly the difficulty in balancing ground clearance, trafficability, and overall stability in crawler chassis, along with their relatively limited operational versatility, this paper proposes the design of a gantry-type high-clearance crawler chassis equipped with a longitudinal center-of-gravity adjustment device. A dual-motor-driven crawler system is implemented to increase driving efficiency, while the gantry frame structure provides increased ground clearance to meet the requirements for interrow operations in tall-stem crops. Furthermore, a longitudinal center-of-gravity adjustment device is designed to actively regulate the relative position between the vehicle frame and the crawler system under various working conditions, such as slope traversal, trench crossing, and step climbing, thereby significantly improving the anti-overturning performance of the chassis. Additionally, an implement pose adjustment mechanism is incorporated to enable compatibility with various agricultural implements and facilitate precision interrow operations through vertical and horizontal positional control.

To achieve the design objectives and validate the performance, the overall system scheme was first designed. A kinematic model of the power chassis was subsequently established, and kinematic analyses were carried out for key working conditions such as slope climbing, contour driving, obstacle crossing, and trench straddling. A virtual prototype of the power chassis was developed using the multibody dynamics software RecurDyn, and dynamic simulations were performed. Finally, a physical prototype was developed, and the driving performance of the chassis was verified through field tests under typical operating conditions.

## 2 Materials and methods

### 2.1 Overall design of the machine

#### 2.1.1 Design objectives

The primary objective of the high-clearance crawler chassis design is to enhance ground clearance while ensuring trafficability and operational stability in complex hilly and mountainous terrain. Additionally, the chassis must be compatible with and capable of mounting various agricultural implements to meet the specific planting requirements of tea and fruit orchards in such regions. The specific design objectives are outlined as follows: (1) To enable cross-row operation, the ground clearance of the chassis shall be no less than 1500mm. (2) To provide a wide range of speed regulation to accommodate varying operational demands under different working conditions. (3) While maintaining high ground clearance, the chassis layout shall be optimized to achieve a lower center of gravity. A longitudinal adjustment mechanism for the center of gravity shall be incorporated to enhance stability when operating on complex terrain. (4) An agricultural implement pose adjustment device with high structural adaptability shall be designed to fulfill diverse agronomic requirements.

#### 2.1.2 Overall machine structure

**Figure 1:**
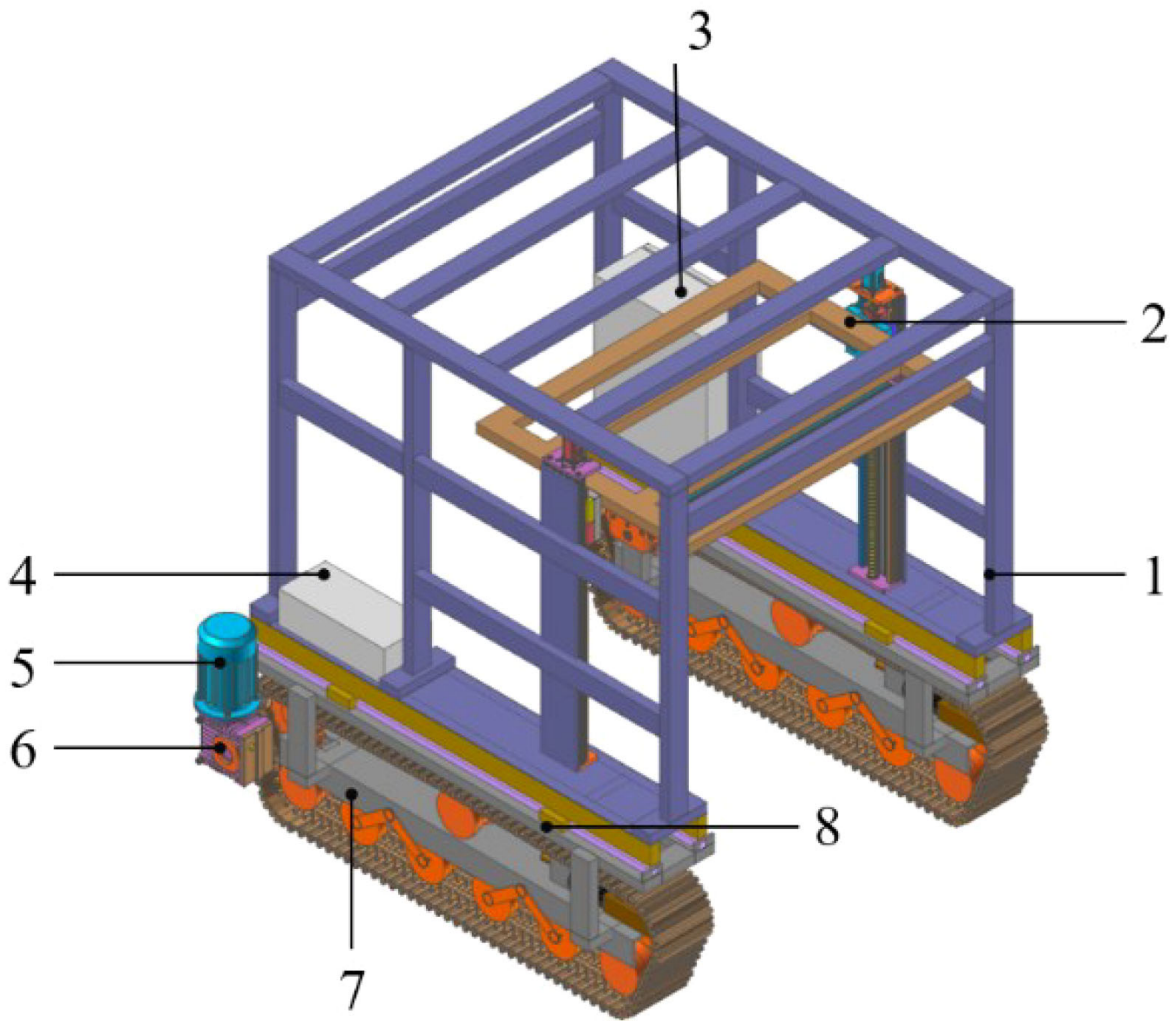
Schematic diagram of the structural layout of the whole machine. 1, frame; 2, agricultural implement position and orientation adjustment system; 3, control cabinet; 4, lithium iron phosphate battery; 5, driving motor; 6, gear reduce; 7, Crawler traveling system; 8, The Longitudinal center-of-gravity adjustment device.

The high-clearance crawler chassis primarily consists of the frame, crawler traveling system, battery pack, drive motor, reducer, controller, longitudinal center-of-gravity adjustment device, and agricultural implement position adjustment device. To achieve the required ground clearance for specific operational conditions, a gantry-type frame structure is adopted. The crawler traveling system, which serves as the driving unit of the chassis, comprises driving wheels, guide wheels, supporting wheels, tensioning devices, and rubber tracks. The drive motor and reducer are integrated externally to the track drive system. The reducer is flange-connected to the driving wheel, transmitting power to the track system and providing propulsion for the entire machine. The front section of the frame interior is equipped with an agricultural implement pose adjustment device, while the lithium iron phosphate battery pack is mounted on the longitudinal beams at the rear to minimize longitudinal shift in the center of gravity. A rail-slider type longitudinal center-of-gravity adjustment device is implemented between the upper frame and the crawler traveling system. This device allows dynamic redistribution of the center of gravity along the longitudinal axis when operating on complex terrain, thereby significantly improving the driving stability of the chassis. The overall structure of the machine is shown in **Figure 1** and **Table 1**.

**Table 1:** Structural and performance parameters of the crawler chassis.

Parameters	Value
Overall machine dimensions (length) × (width) × (height)/(mm × mm × mm)	1700×1600×1800
Total machine mass/kg	600
Working width/mm	1100
Driving speed/km·h^-^¹	0~5.4
Maximum ground clearance/mm	1550
Track contact length/mm	1500
Maximum climbing angle/°	35
Maximum trench-crossing width/mm	750
Maximum obstacle-crossing height/mm	300

#### 2.1.3 Critical component design

##### 2.1.3.1 Drive system

**Figure 2:**
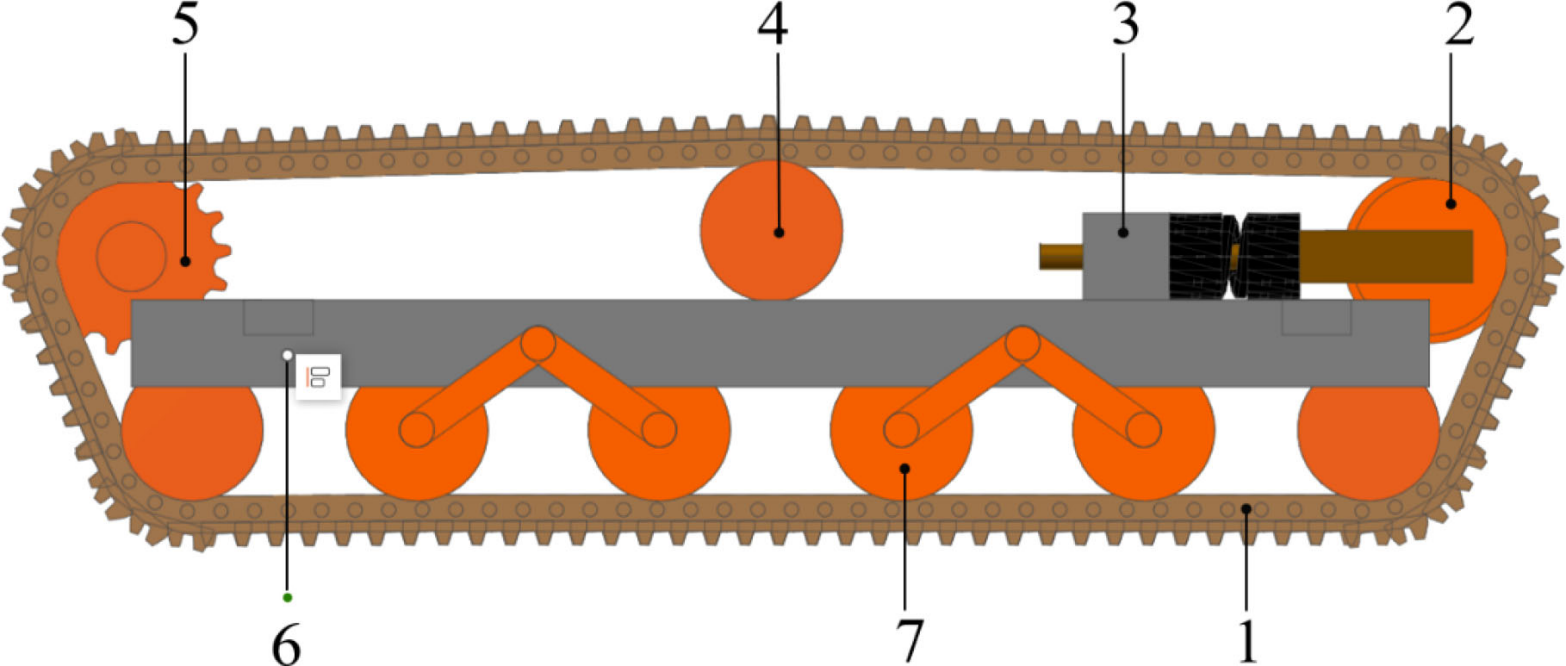
Crawler traveling system. 1, rubber track; 2, guide wheel; 3, tensioning device; 4, carrier roller; 5, drive wheel; 6, chassis frame; 7, bearing roller.

The crawler traveling system, as the driving unit of the crawler chassis, plays a critical role in determining its overall traversing performance. This system consists of a drive sprocket, idler wheel, carrier rollers, track rollers, frame, tensioning device, and track assembly. Given that crawler chassis are primarily deployed in complex terrains such as hilly and mountainous areas, rubber tracks with a ground contact length of 1500mm and a width of 230mm are selected for their superior terrain adaptability, lighter weight, improved traction, and reduced ground damage. To further enhance the contour-following ability of the tracks on the ground and increase the contact area of the tracks with the ground, a combined carrier rollers scheme is adopted. (**Figure 2**).

In hilly and mountainous regions characterized by complex terrain and fragmented plots, agricultural operations necessitate frequent turning maneuvers. To meet this requirement, this drive scheme adopts a dual-motor distributed drive system. In this system, two independent 2kW DC motors are installed on the left and right sides, and the power is transmitted to the driving wheels through a reducer with a ratio of 20:1 (Wang et al., 2022). This configuration enables independent speed control of the left and right tracks, allowing differential movement at any ratio. Steering is achieved by regulating the speed difference between the two tracks, facilitating pivot turning. The independently controlled tracks enhance performance on challenging slopes and reduce slippage risks. Moreover, this system eliminates the need for central differentials, drive shafts, and steering linkages, thereby minimizing potential failure points and significantly improving the overall reliability of the crawler chassis.

##### 2.1.3.2 Agricultural implement position and orientation adjustment system

**Figure 3:**
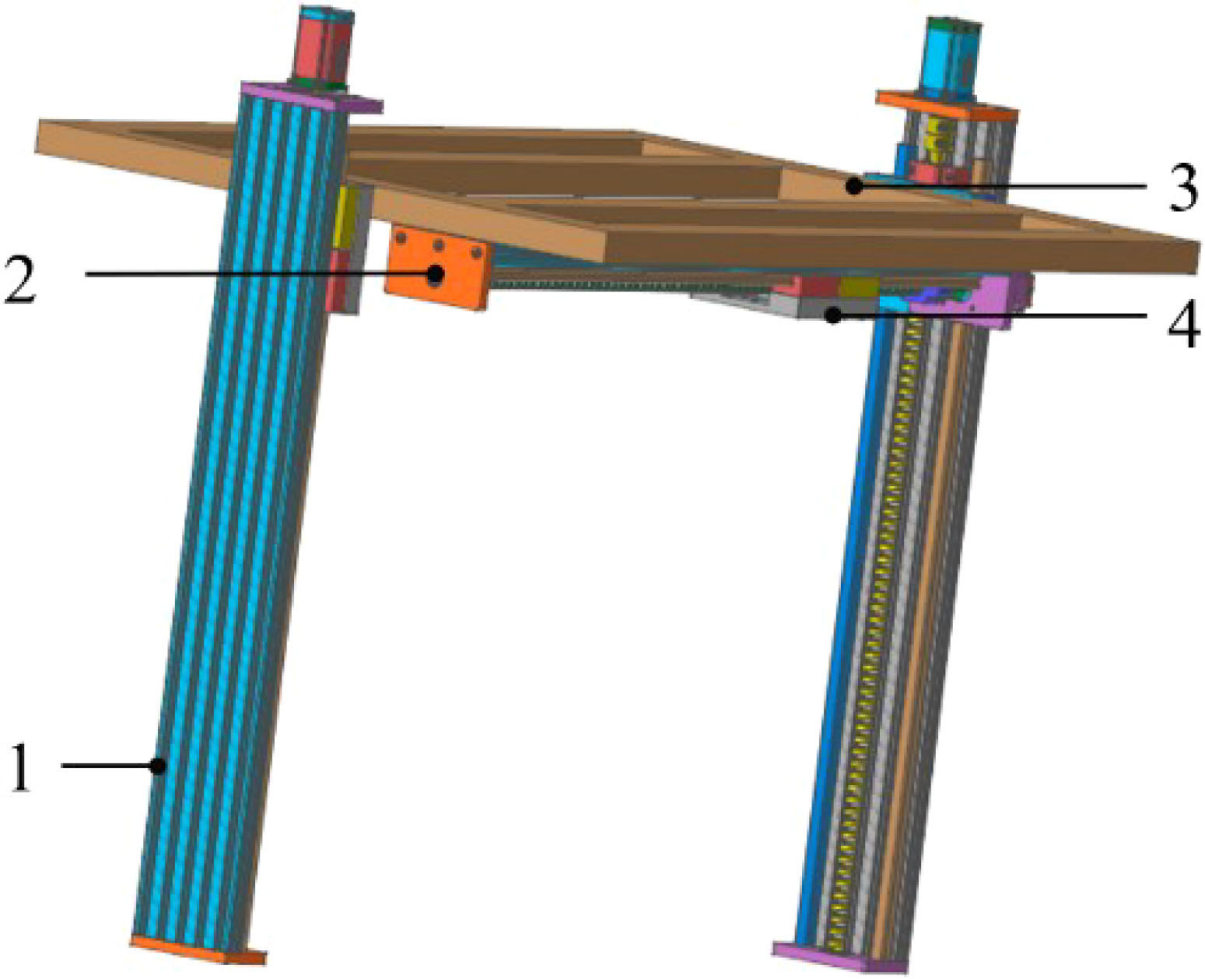
A device for adjusting the position and posture of agriculture tools. 1, spiral lifting drive mechanism; 2, translation drive mechanism; 3, work platform; 4, implement mounting base.

To enhance the versatility and operational flexibility of the crawler chassis, an agricultural implement pose adjustment device has been designed. Its core function is to enable rapid mounting and interchangeability of implements, along with precise vertical and horizontal positioning, thereby supporting precision farming operations across various crops. Specifically, to achieve reliable positioning and stable operation of the work platform at multiple heights, the system incorporates two symmetrically installed guide rail screw sliding tables with a stroke of 800mm and a load capacity of 1500N as the lifting drive mechanism. These are mounted on the vertical columns on both sides of the gantry frame. The work platform is fixed to the sliding tables via mounting brackets on each side. A stepper motor drives the two screw mechanisms synchronously, enabling the platform to ascend and descend smoothly. The dual sliding table design offers high load-bearing capacity and structural stability, ensuring vertical motion without tilting or shaking. Furthermore, the mechanism provides self-locking in power-off or stationary states, allowing the platform to remain securely positioned at any height within its range to perform operational tasks. To ensure accuracy in cross-row operation, a guide rail screw sliding table with a stroke of 1000mm and a load capacity of 1000N is adopted as the horizontal drive unit. It is installed on the work platform framework and used to mount different implements while enabling their lateral movement for aligned and precise field operations. (**Figure 3**).

##### 2.1.3.3 Longitudinal center-of-gravity adjustment device

**Figure 4:**
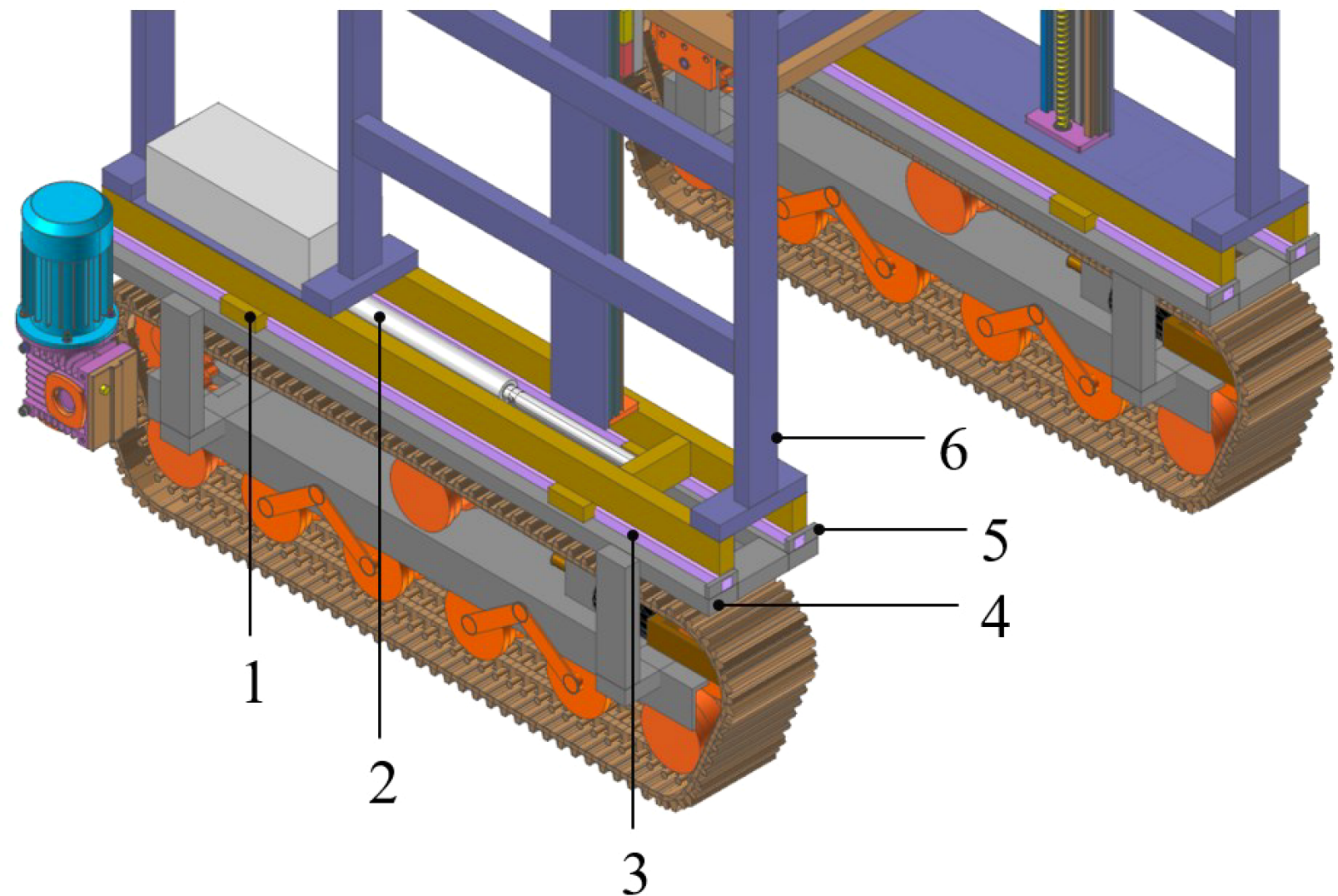
Longitudinal center-of-gravity adjustment device 1, ball bearing slider; 2, push rod; 3, linear guide rail; 4, load-bearing frame of the crawler traveling system; 5, limit block; 6, frame.

To improve the longitudinal stability and terrain adaptability of the crawler chassis, a longitudinal center-of-gravity adjustment device consisting of a linear motion system and an electric push rod is designed. The linear motion system incorporates high-strength linear guides and ball sliders characterized by low friction and high guiding accuracy. The guide rail is mounted on the load-bearing frame of the crawler traveling system unit using high-strength bolts. Based on the maximum slope of the designed chassis (35°) and the actual measured center of gravity height of the chassis (480 mm from the ground), the calculated center of gravity offset (the adjustment range required by the center of gravity adjustment device) is 336 mm (Han et al., 2022b). To ensure a certain margin, it is finally determined to be 350 mm. Therefore, two sliders are fixed 350 mm from each end of the longitudinal beam at the bottom of the vehicle frame, ensuring continuous engagement between the frame and the crawler traveling system during relative motion. Each set of linear motion components comprising one guide rail and two matching sliders is arranged symmetrically. This configuration restricts the longitudinal displacement of the frame relative to the crawler system, supports all vertical loads from the frame and attached equipment, and maintains the lateral strength and overall torsional rigidity of the chassis. As the driving part of the center of gravity adjustment system, the electric push rod is hinged at both ends to the frame and crawler traveling system, and the hinge device allows for a small range of deflection. The hinge location is determined through kinematic analysis to ensure symmetric forward and backward adjustment strokes of the frame. (**Figure 4**).

The longitudinal center-of-gravity adjustment device enables active regulation of the vertical center-of-gravity position through a control strategy based on “real-time monitoring, intelligent decision-making, and precise execution”. A chassis-mounted tilt sensor continuously monitors key attitude parameters during operation, including the terrain slope and chassis pitch angle. These data are converted via an RS485 interface module and transmitted to the main STM32 controller. Upon receiving the sensor signals, the controller compares the current attitude parameters with predefined critical thresholds. If the detected values exceed these thresholds, corresponding control commands are generated and sent to the electric actuator driver. This actuates telescopic movement in the linear actuator, altering the relative position between the frame and the undercarriage, thereby achieving precise adjustment of the center of gravity.

#### 2.1.4 Operating principle of the whole machine

**Figure 5:**
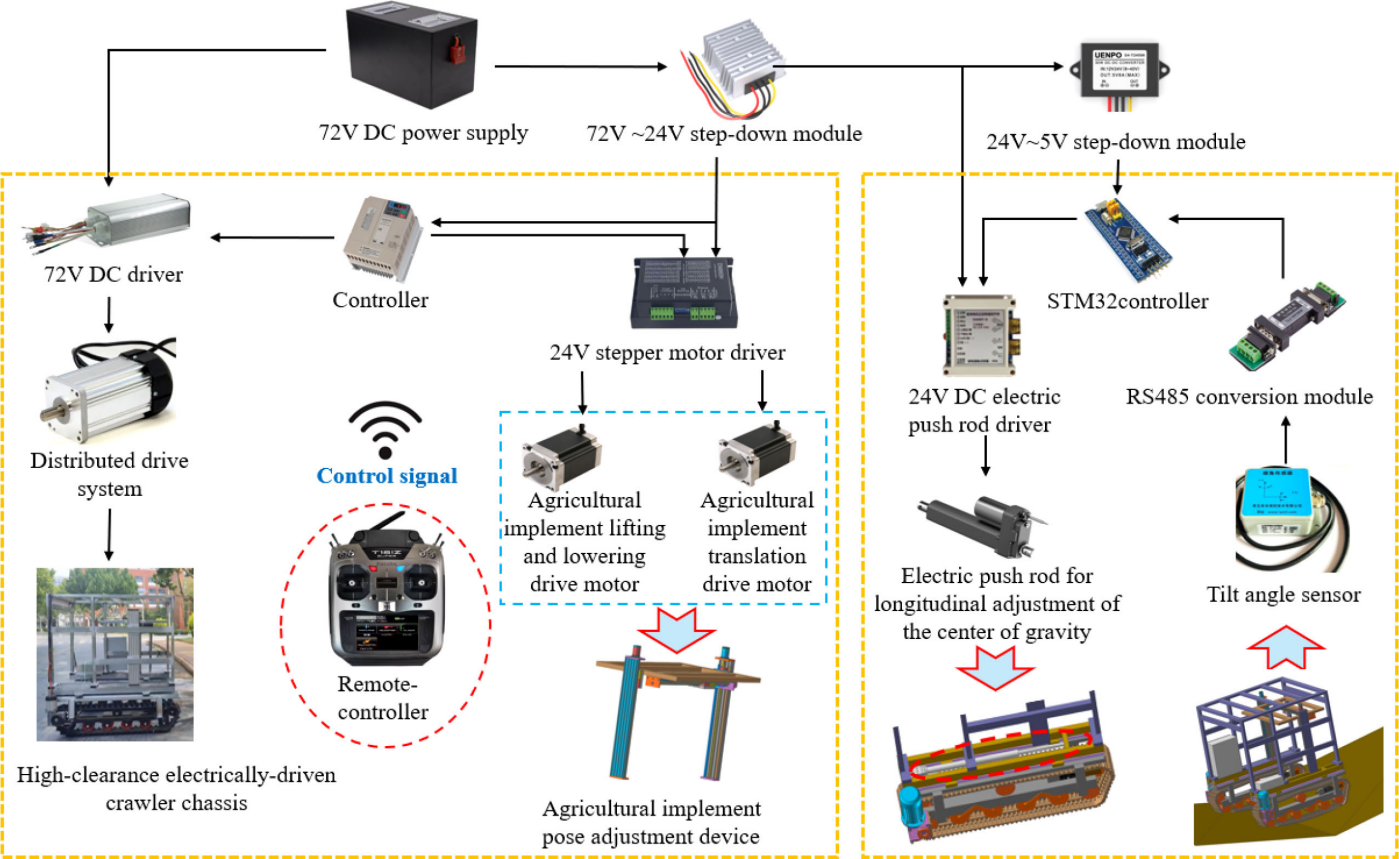
Operating principle of the whole machine.

The chassis employs a dual-motor distributed drive system, allowing wireless remote control of propulsion, steering, and braking. The maximum remote control distance is 100 meters. During straight-line travel, the onboard controller commands both drive motors to operate at identical speeds. The power is transmitted through reducers to propel the crawler traveling mechanisms. For steering, the controller differentially regulates the rotational speeds of the two motors. When the motors counterrotate at equal speeds, the crawler chassis executes a pivot turn. The longitudinal center-of-gravity adjustment device modulates the relative position between the vehicle frame and the crawler traveling system by varying the extended length of the electric push rod, thereby improving longitudinal driving stability. The agricultural implement pose adjustment device is designed to accommodate implements suited to various agronomic needs. It enables vertical lifting and positioning of implements at multiple heights, as well as horizontal movement across crop rows, supporting high-precision interrow operations. The operating principle of the complete machine is illustrated in **Figure 5**.

### 2.2 Theoretical analysis of the overall machine driving performance

The high-clearance crawler chassis uses a gantry-type frame, which elevates the center of gravity of the entire machine. To prevent overturning under complex working conditions, it is essential to perform a stability analysis, establish a critical overturning moment equation, and examine the relationship between the driving performance of the chassis and its structural parameters under various unstructured terrain conditions. Given that the chassis employs a crawler-type travel mechanism and typically operates at low speeds during normal travel and obstacle negotiation, secondary factors such as air resistance and mechanical transmission efficiency losses are neglected in the stability analysis (Chen et al., 2024).

#### 2.2.1 Analysis of the driving stability on longitudinal slopes

**Figure 6:**
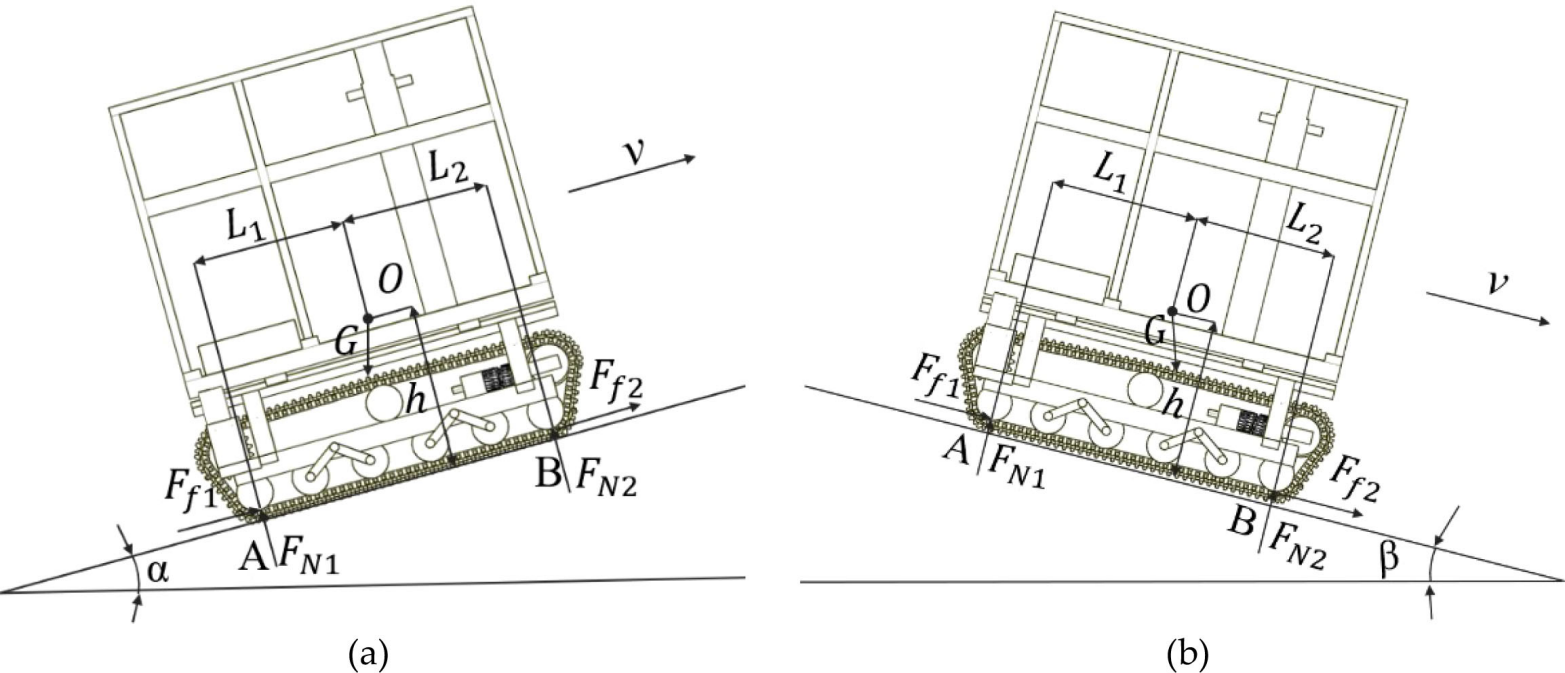
Stability analysis of driving on longitudinal slopes. **(a)** Climbing condition; **(b)** Downhill condition.

When the crawler chassis climbs a slope longitudinally at a constant speed, it remains in a state of mechanical equilibrium, that is, the resultant external force acting on it is zero. Neglecting the effects of air resistance and mechanical transmission efficiency losses, the main forces considered include gravity, the operational friction force, and the reaction force from the sloping ground (**Figure 6a**). The moment equilibrium equation is formulated with respect to support point A. as shown in Equation 1. (**Figure 6**).

(1) $$\left\{ \begin{array}{l} F_{N1}+F_{N2}=G\cos\alpha \\ F_{f1}+F_{f2}=G\sin\alpha \\ Gh\sin\alpha +F_{N2}(L_{1}+L_{2})=GL_{1}\cos\alpha \end{array}\right.$$

When the crawler chassis reaches the limit state of longitudinal uphill tipping, the ground reaction force at support point B decreases to zero. On the basis of these conditions, the longitudinal uphill tipping limit angle α of the chassis can be determined as shown in Equation 2.

(2) $$\alpha =\arctan\frac{L_{1}}{h}$$

Similarly, a force analysis is conducted for the longitudinal downhill condition of the crawler chassis (**Figure 6b**). By taking moments about support point B, the moment equilibrium equation is established, from which the longitudinal downhill limit tipping angle β of the chassis is derived as shown in Equation 3.

(3) $$\beta =\arctan\frac{L_{2}}{h}$$

Analysis indicates that the ultimate longitudinal tipping angle is primarily determined by the vertical height of the machine’s center of gravity above the ground and its horizontal distance to the support point. When the vertical height of the center of gravity remains constant, increasing the horizontal distance to the support point effectively increases the longitudinal tipping limit angle. The longitudinal center-of-gravity adjustment device designed in this study regulates the overall center of gravity by altering the longitudinal relative position between the frame and the crawler traveling mechanism. This adjustment alters the horizontal distance from the center of gravity to the support point, thereby increasing the longitudinal tipping limit angle and enhancing the longitudinal trafficability of the crawler chassis.

#### 2.2.2 Analysis of driving stability on lateral sloping terrain

**Figure 7:**
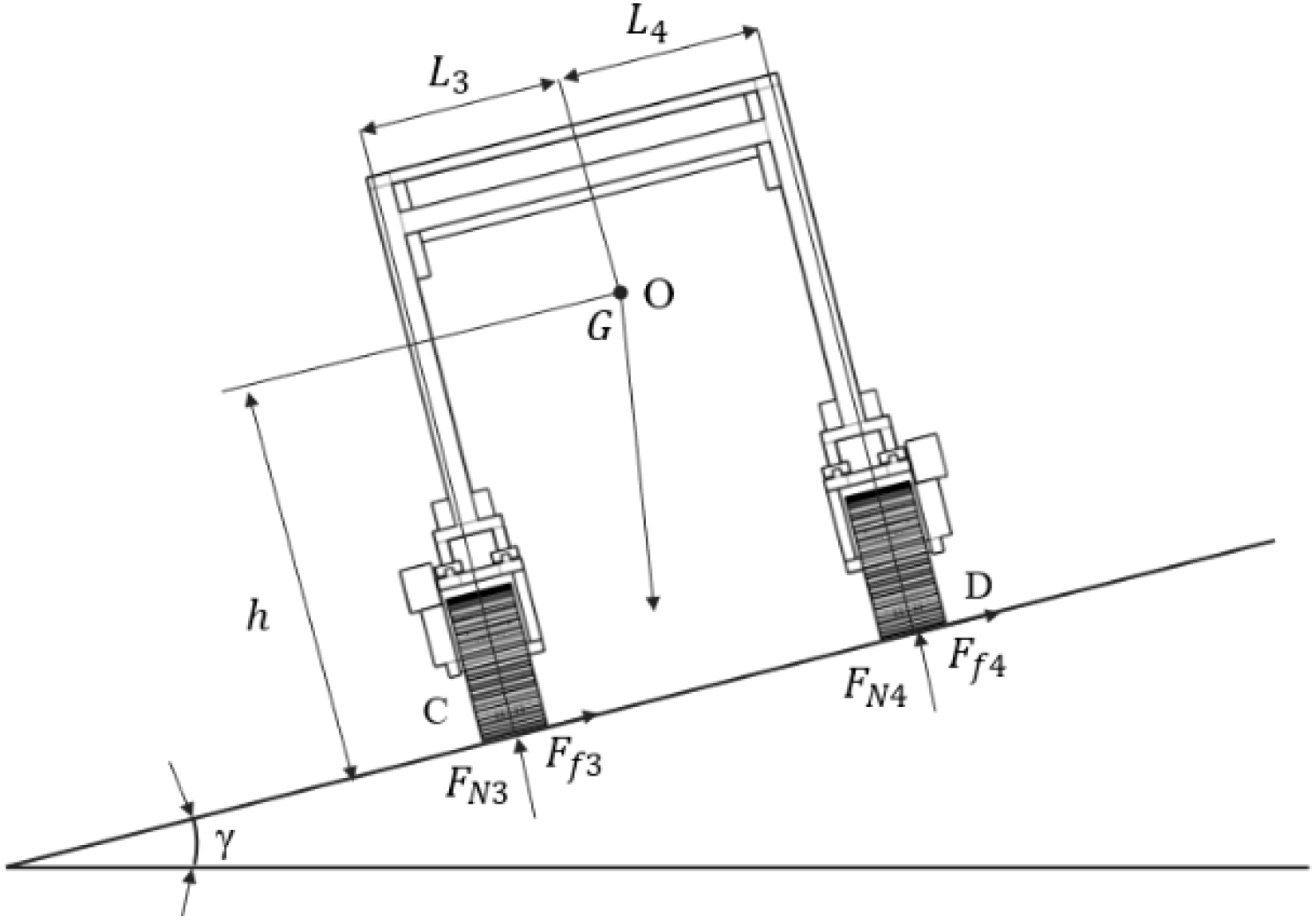
Driving conditions on lateral-slope terrain.

Lateral slope stability describes the ability of a crawler chassis to resist overturning when it operates along contour lines. Using the slope support reaction as the evaluation criterion, the lateral ultimate tipping angle can be analyzed. When the crawler chassis moves at a constant speed on a lateral slope, it remains in equilibrium with a net force of zero. Neglecting the influence of air resistance and mechanical transmission losses, the main forces acting on the chassis include gravity, friction generated during motion, and ground reaction forces from the slope (**Figure 7**). The moment equilibrium equation is established by taking moments about support point C. as shown in Equation 4. (**Figure 7**).

(4) $$\left\{ \begin{array}{l} F_{N3}+F_{N4}=G\cos\gamma \\ F_{f3}+F_{f4}=G\sin\gamma \\ Gh\sin\gamma +F_{N4}(L_{3}+L_{4})=GL_{3}\cos\gamma \end{array}\right.$$

When the crawler chassis reaches the limit of lateral tipping during slope traversal, the ground reaction force at point D becomes zero. Under these conditions, the lateral slope tipping limit angle γ of the chassis can be determined as shown in Equation 5.

(5) $$\gamma =\arctan\frac{L_{3}}{h}$$

Moreover, the amount of lateral slip during cross-slope operation is also a critical indicator of driving stability. On the basis of an analysis of the lateral forces acting on the crawler chassis, the mechanical equilibrium conditions required to prevent sliding are established. This enables the determination of the critical angle $\gamma^{1}$ at which the crawler chassis can travel on a lateral slope without slipping as shown in Equation 6.

(6) $$F_{f3}+F_{f4}=G\sin\gamma_{1}\leq \mu G\cos\gamma_{1}$$

In conclusion, the lateral driving stability of the crawler chassis is influenced by both rollover and slippage. Analysis based on equations (5) and (6) indicates that the antirollover performance on lateral slopes is governed by the vertical height of the machine’s center of gravity above the ground and the horizontal distance from the center of gravity to the lateral support points. A lower vertical center of gravity and a greater horizontal distance to the support points result in a larger ultimate rollover angle. Moreover, resistance to lateral slippage depends on the ground adhesion coefficient and the grip characteristics of the tracks. A higher ground adhesion coefficient, coupled with improved track grip, increases slippage resistance on lateral slopes.

#### 2.2.3 Analysis of obstacle-crossing performance

The primary advantage of the crawler chassis is its superior ability to traverse obstacles on complex or unstructured terrain. To analyze the relationship between the chassis’ trafficability when it crosses vertical steps and its structural parameters, the kinematic process of obstacle crossing is examined by decomposing its motion stages, and corresponding mechanical equilibrium equations are established.

The process by which a crawler chassis crosses a vertical step obstacle can be divided into three interrelated stages: the contact and ascent stage, the chassis traversal stage, and the descent and contact stage.

During the contact and ascent phase, the leading edge of the crawler chassis contacts the rim of the vertical step. Driving torque is transmitted through the tracks to the step, generating an upward lifting force. This causes the center of mass of the chassis to increase and initiate rotation around the contact point of the rear tracks. Throughout this process, the position of the center of mass shifts considerably in both the vertical direction and the horizontal direction. To ensure sufficient lifting force, the height ${\text{h}}_{1}$​ of the guide wheel’s center of rotation must exceed the step height ${\text{h}}_{0}$​.

**Figure 8:**
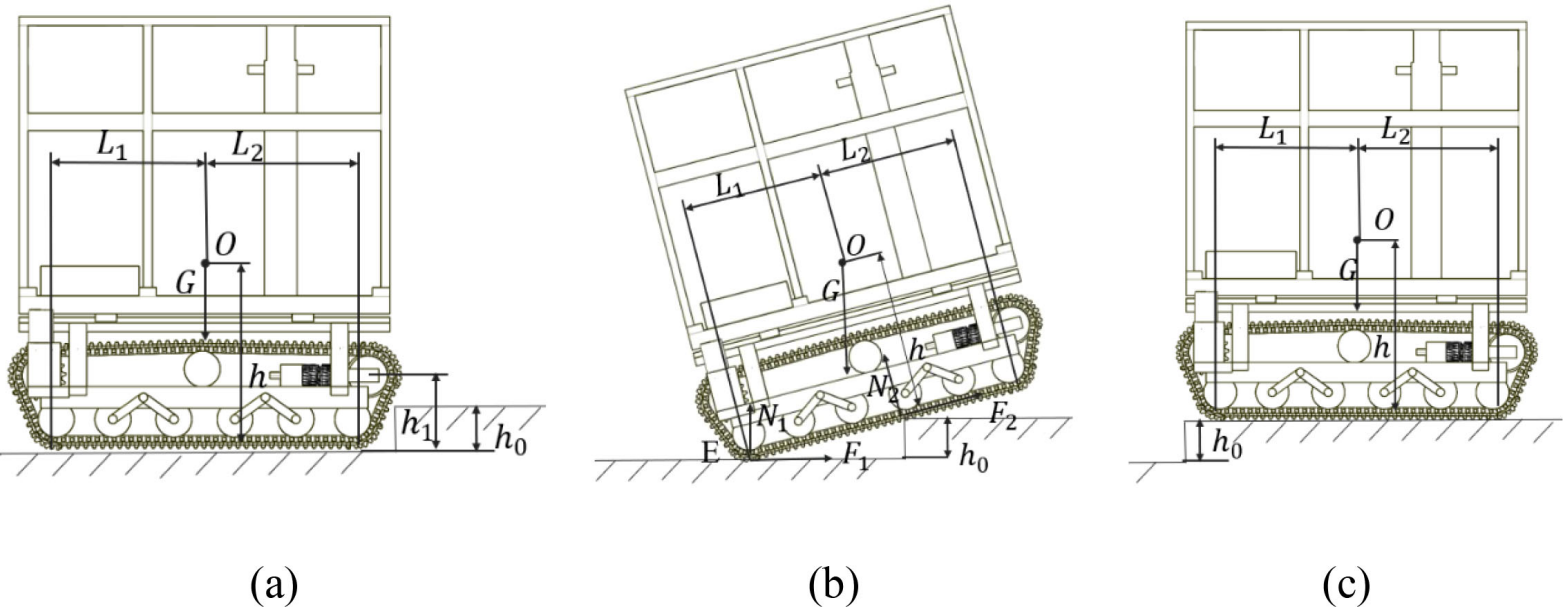
Obstacle-crossing working conditions. **(a)** stage 1; **(b)** stage 2; **(c)** stage 3.

During the chassis traversal stage, after the front idler wheels establish full contact with the upper edge of the vertical step, the chassis begins to be pulled upward. Throughout this process, the tracks rotate around the support point while advancing until the rear section of the tracks lifts from the lower road surface. At this stage, the chassis approaches a critical tipping state. When the ground reaction force is used as an evaluation criterion, the limiting condition for crossing the vertical step is reached when the center of gravity of the vehicle aligns vertically with the edge of the step (Lin et al., 2022). At this point, the main forces acting on the crawler chassis include its own weight, the support and traction forces applied by the ground on the rear support wheels, and the support and traction forces exerted by the vertical step on the engaged track segments (**Figure 8**).

On the basis of the force analysis in the second stage, the moment equilibrium equation is established by taking moments about point E, which is the contact point between the rear end of the crawler and the ground as shown in Equation 7:

(7) $$N_{2}(L_{1}-h\tan\theta )+hG\sin\theta -L_{1}G\cos\theta =0$$

The critical pitch angle is reached when the reaction force from the step on the crawler track decreases to zero. On the basis of equation (7), the critical pitch angle for the crawler chassis crossing a vertical step can be determined as follows as shown in Equation 8:

(8) $$\theta_{\text{max}}=\arctan(\frac{L_{1}}{h})$$

On the basis of the functional relationship between the critical pitch angle and the surmountable vertical step height, the maximum height of a vertical step that can be crossed can be determined as follows as shown in Equation 9:

(9) $$h_{0}(\theta )=(L_{1}-h\tan\theta )\sin\theta$$

By incorporating the key parameters identified during the initial phase, the maximum traversable vertical step height can be expressed as follows as shown in Equation 10:

(10) $$h_{0}=\min(h_{1},h_{0}(\theta ))$$

During the descent and contact stage, the crawler chassis begins to descend while it continues to move forward until the rear idler wheels contact the ground on the upper side of the step. The main factor affecting stability in this phase is the impact force generated when the front end of the chassis touches the ground.

Integration of the three stages of vertical step crossing described above reveals that during the contact and climbing stage, the height at which the driving wheel is installed directly determines the height at which the track contacts the vertical step, thereby influencing the maximum surmountable step height. During the chassis traversal stage, the ratio of the height of the center of gravity to its horizontal distance from the rear idler wheel affects the vehicle’s ability to cross the step. In the descent and contact stage, the primary concern is the impact generated upon landing; hence, the resulting stability and safety must be considered. The maximum vertical step height that the crawler chassis can safely cross must simultaneously satisfy the constraints derived from all three stages. The crawler travel system designed in this study employs trapezoidal tracks, which provide a relatively high installation position for the driving wheels. In addition, the longitudinally adjustable center-of-gravity mechanism can alter the position of the overall center of gravity during step climbing, thereby increasing the maximum achievable pitch angle. Compared with a conventional crawler chassis without a longitudinal center-of-gravity adjustment device, this design enhances the ability of the vehicle to traverse taller steps.

#### 2.2.4 Analysis of trench-crossing performance

As trenches are common obstacles in hilly and mountainous regions, trench-crossing ability is an important indicator for evaluating the overall trafficability of chassis systems. To investigate the relationship between the trench-crossing performance of the crawler chassis and its structural parameters, the trench traversal process was analyzed.

**Figure 9:**
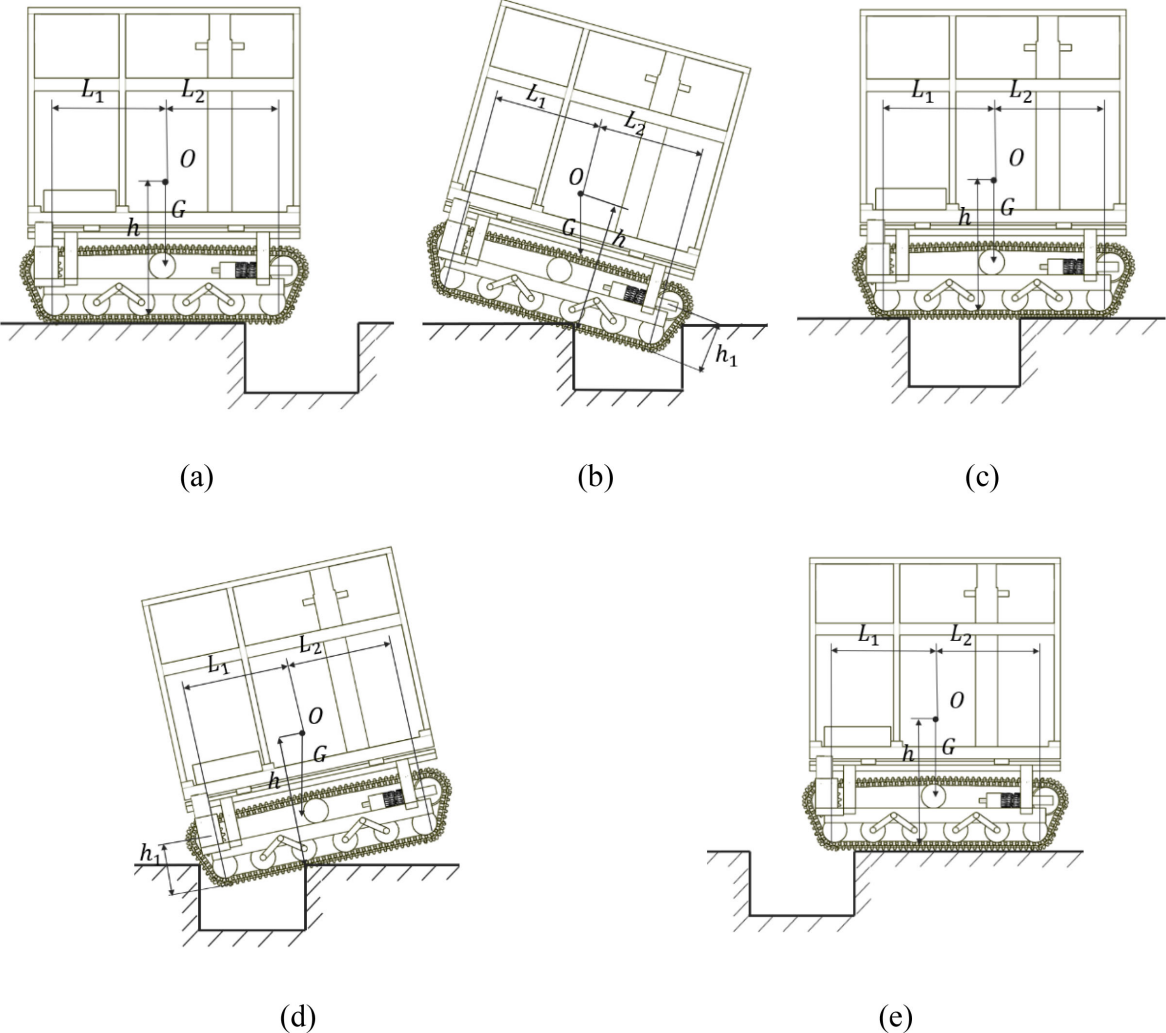
Trench–crossing working conditions. **(a)** stage 1; **(b)** stage 2; **(c)** stage 3; **(d)** stage 4; and **(e)** stage 5.

The trench-crossing process of a crawler chassis constitutes a continuous multistage operation (**Figure 9**). In the initial stage, the crawler approaches the trench, leading to a loss of contact between the front idler wheels and the ground. This reduces the front support force and introduces pitching motion. During the second stage, only a limited number of track rollers remain in ground contact. If the vertical projection of the center of mass of the vehicle moves beyond the front supporting point, the chassis becomes susceptible to forward tipping. The third stage involves the impact between the front crawler section and the opposite slope of the trench. This impact load counteracts the pitching motion and allows the front end to cross the trench. In the fourth stage, the rear end of the crawler disengages from the edge of the trench, with only a few rear track rollers maintaining ground contact. If the vertical projection of the center of mass extends beyond the rear supporting point, the risk of backward tipping increases significantly. The final stage is marked by renewed ground contact with the tracks under impact loading, completing the trench-crossing process. (**Figure 9**).

Unstable states occur in the second and fourth stages when the crawler chassis crosses a trench. When the position of the center of gravity coincides with the vertical wall at the edge of the trench, the crawler chassis reaches the tipping angle limit. To ensure that the crawler chassis crosses the trench safely, neither end of the crawler should fully enter the trench during both forward and backward tipping in the trench-crossing process as shown in Equations 11, 12. That is,

(11) $$\left\{ \begin{array}{l} H_{1}=\sqrt{{\left(L_{1}-h\tan\epsilon \right)}^{2}+h_{1}2}\\ H_{2}=\sqrt{{\left(L_{2}-h\tan\epsilon \right)}^{2}+h_{1}2} \end{array}\right.$$

(12) $$H=\min(H_{1},H_{2})$$

The trench-crossing capability of a crawler chassis is influenced by multiple factors, including the position of its center of mass, travel speed, and mass distribution. The vertical and longitudinal locations of the center of gravity govern the maximum pitch angle achievable during trench traversal. The travel speed and mass distribution affect the relative position between the crawler and the edge of the trench when the critical pitch angle is reached, thereby influencing the overall trench negotiation performance. The longitudinal center-of-gravity adjustment device designed in this study modulates the mass distribution in real time during trench crossing. This adjustment increases the limiting pitch angle, reduces the rate of pitch variation, and enhances the trench trafficability of the crawler chassis.

## 3 Results and discussion

### 3.1 Simulation of the overall machine’s driving stability

To systematically evaluate the stability and trafficability of the crawler chassis under complex working conditions, validate the theoretical conclusions, and verify the effectiveness of the longitudinal center-of-gravity adjustment device, a virtual prototype of the high-clearance crawler chassis was developed using the multibody dynamics software RecurDyn (Wang et al., 2024). Driving performance simulations were subsequently conducted. The bottom plane of the vehicle frame was defined as the coordinate origin. In accordance with Bekker’s terrain mechanics theory, two typical terrain models with sinkage and shear characteristics representing clay and sandy soils were established to simulate real-world operating environments (Jin et al., 2022). The specific soil parameters are provided in **Table 2** (Zhang et al., 2024). In order to ensure the accuracy of the simulation results, the simulation step size is set to 1/60 second. Kinematic simulations were performed under multiple operational conditions, and the corresponding driving state parameters of the chassis were extracted for analysis (Coneo et al., 2025). (**Table 2**).

**Table 2:** Parameters of the pavement model.

Pavement types	Terrain stiffness(k_c)/(N·mm-n-1)	Terrain stiff ness(k_phi)/(N·mm-n-2)	Exponential number(n)	Cohesion/(kPa)	Shearing resistance angle/(°)	Shearing deformation medulus/(mm)
clay	0.4171	0.0219	0.5	0.00414	13	25
sandy soil	0.0419	0.0120	0.7	0.00172	29	25

#### 3.1.1 Simulation of longitudinal sloping terrain trafficability

**Figure 10:**
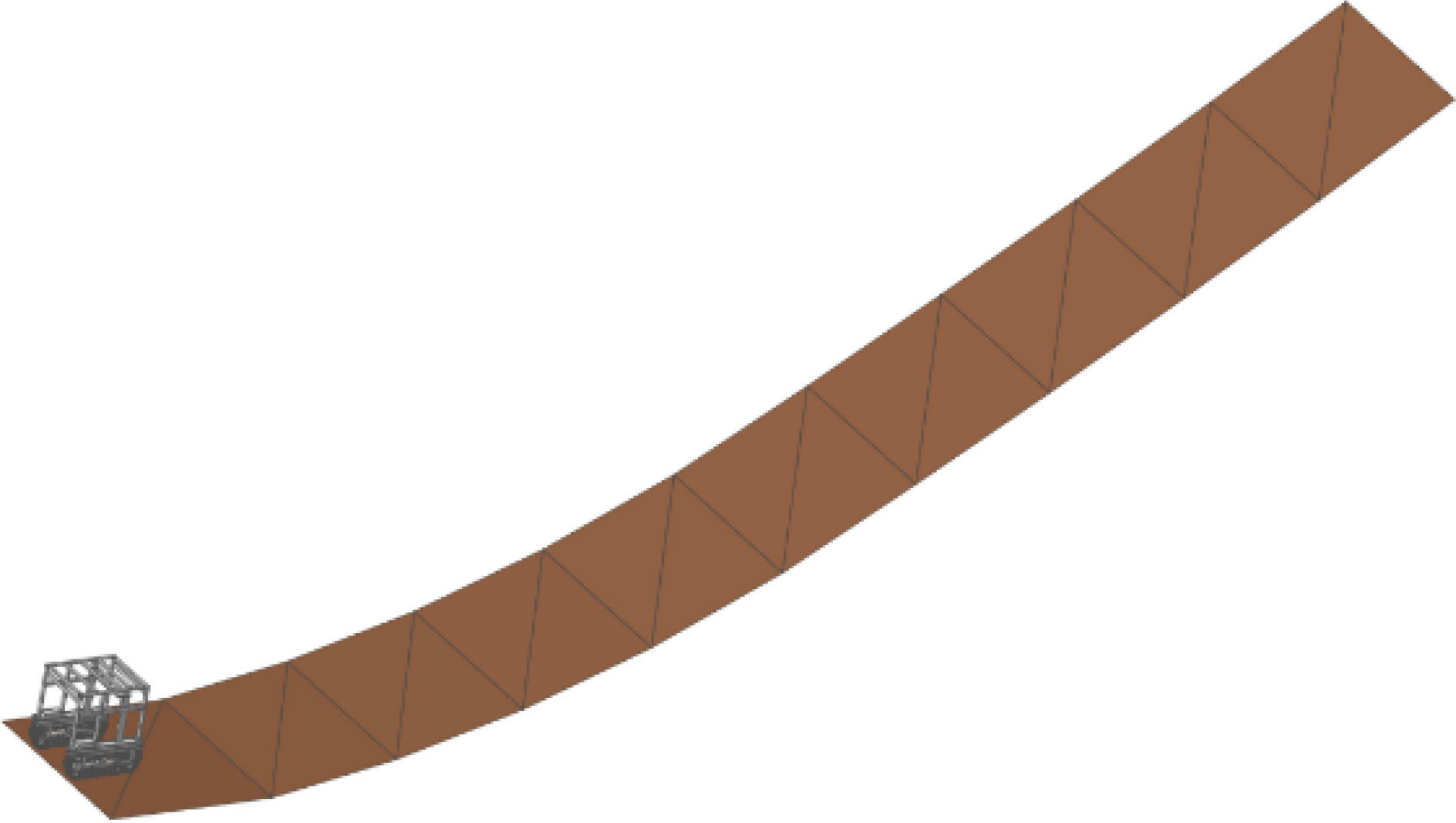
Longitudinal continuous slope terrain model (0~40°).

To evaluate the driving performance of the crawler chassis under typical longitudinal slope conditions in hilly and mountainous terrain, key factors influencing slope trafficability, and assess the effect of the longitudinal center-of-gravity adjustment device on slope-climbing stability, simulations were conducted using continuous slopes with lengths of 5 m and inclinations of 10°, 15°, 20°, 25°, and 30~40° (increasing by 1° beyond 30°). These simulations were performed on both clay and sandy terrain models, which were established on the basis of Bekker’s theory to represent typical soft-soil conditions (**Figure 10**). This range of slope angles comprehensively covers the typical climbing scenarios encountered during operations in such regions. The initial speed of the crawler chassis was set to 0 km/h. On level ground, it accelerated to 3.6 km/h, and after this speed stabilized, it entered the slope section. Comparative simulations were carried out under both terrain types with and without the center-of-gravity adjustment feature. (**Figure 10**).

**Figure 11:**
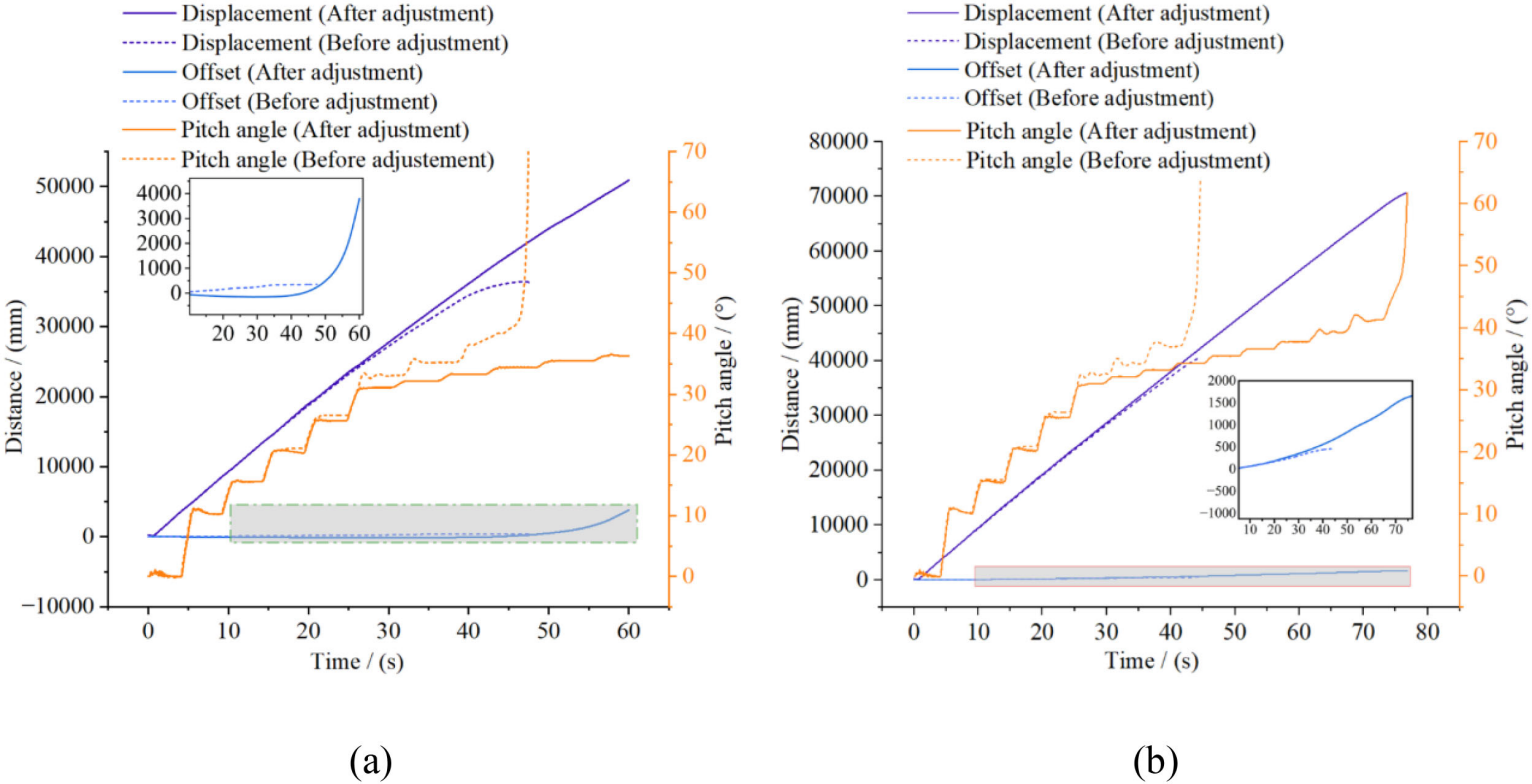
Results of longitudinal uphill climbing simulation. **(a)** Clay working conditions; **(b)** Sandy soil working conditions.

The simulation results presented in **Figure 11** demonstrate that under clay road conditions, the crawler chassis maintained stable operation on slopes up to 25° prior to center-of-gravity adjustment. At 30°, slippage occurred, followed by severe skidding at 32°, accompanied by a rapid increase in the pitch angle, indicating loss of stability and backward tipping. After center-of-gravity adjustment, the chassis remained stable on a 32° slope, with significantly reduced slippage compared with that of the unadjusted configuration. However, a certain degree of deviation was observed during steep slope traversal. Under sandy road conditions, no significant slippage occurred before adjustment. The vehicle lost stability and tipped backward when it entered a 33° slope. After adjustment, the chassis successfully traversed a 33° slope without instability, with backward tipping occurring at 38°. No significant slippage was observed throughout the process.

The longitudinal center-of-gravity adjustment device significantly improves the climbing performance of the chassis on various longitudinal slopes. On clay surfaces, on which slippage is probable, the device primarily functions to reduce skidding and prevent tipping. On sandy surfaces with greater adhesion, the device effectively increases the maximum climbable slope angle. This improvement is attributed to the forward shift in the center of gravity, which not only increases the horizontal distance from the center of gravity to the rear support point—thereby increasing the anti-tipping moment and the ultimate tipping angle—but also increases the ground pressure of the tracks, leading to improved traction. These results validate both the theoretical analysis and the functional design of the longitudinal center-of-gravity adjustment device. (**Figure 11**).

#### 3.1.2 Simulation of driving on contour slopes

**Figure 12:**
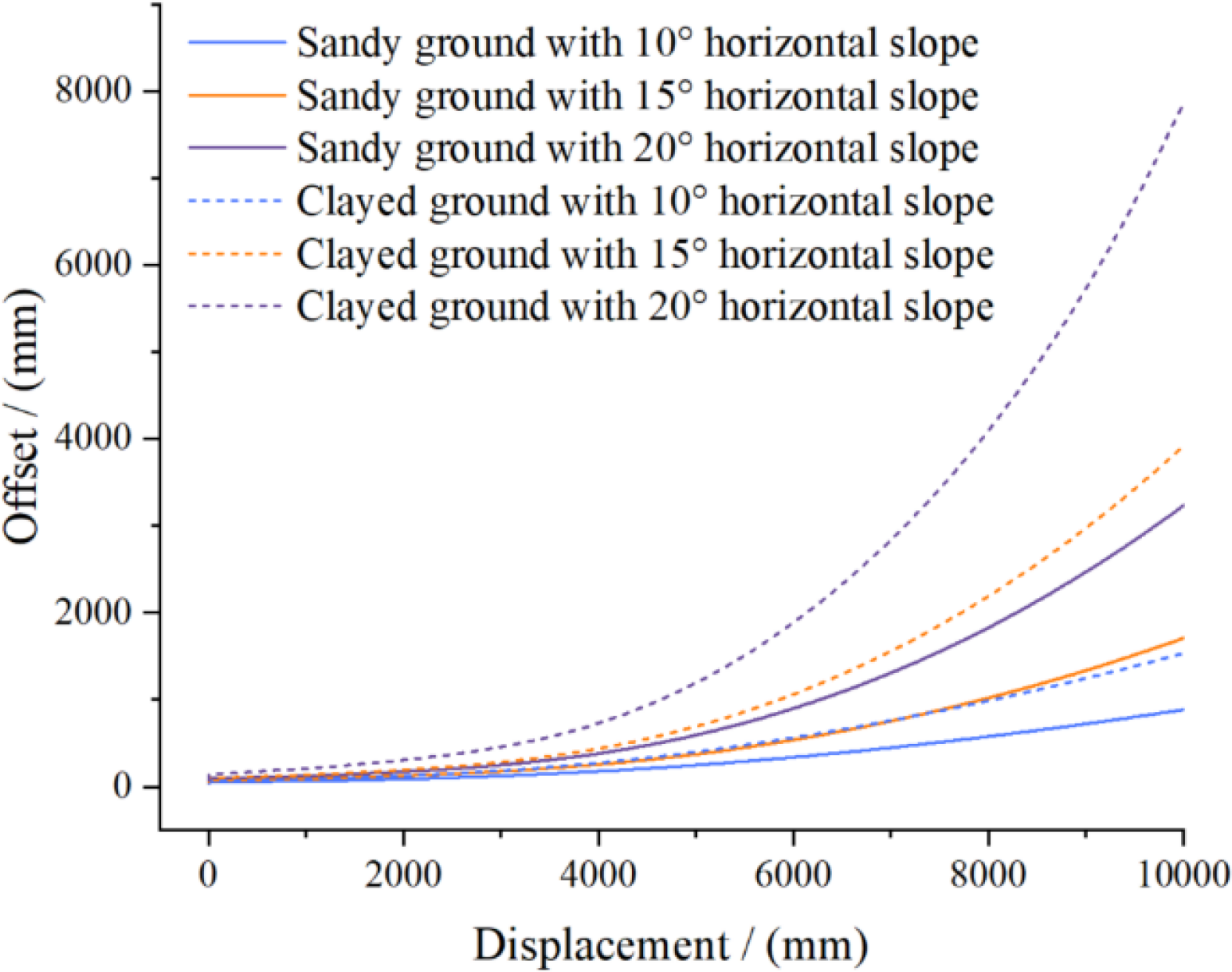
Simulation results of vehicle travel along contour lines on different soil slopes of various inclinations.

To evaluate the lateral stability of the high-clearance crawler chassis on transverse slopes, simulations were conducted on both clay and sandy soil terrain models with slope angles of 10°, 15°, and 20°. The vehicle speed was set to 3.6 km/h. The chassis started from rest on the specified transverse slope, accelerated to the target speed, and then maintained a constant velocity. The simulation results shown in **Figure 12** reveal that the slip rate and yaw rate on clay were significantly greater than those on sandy soil. These instability phenomena intensified with increasing slope angle. This can be attributed to the increased downslope component of gravity at steeper angles, which redistributes the normal load across the slope surface and disrupts the traction balance between the two tracks, thereby exacerbating slippage and yawing. (**Figure 12**).

**Figure 13:**
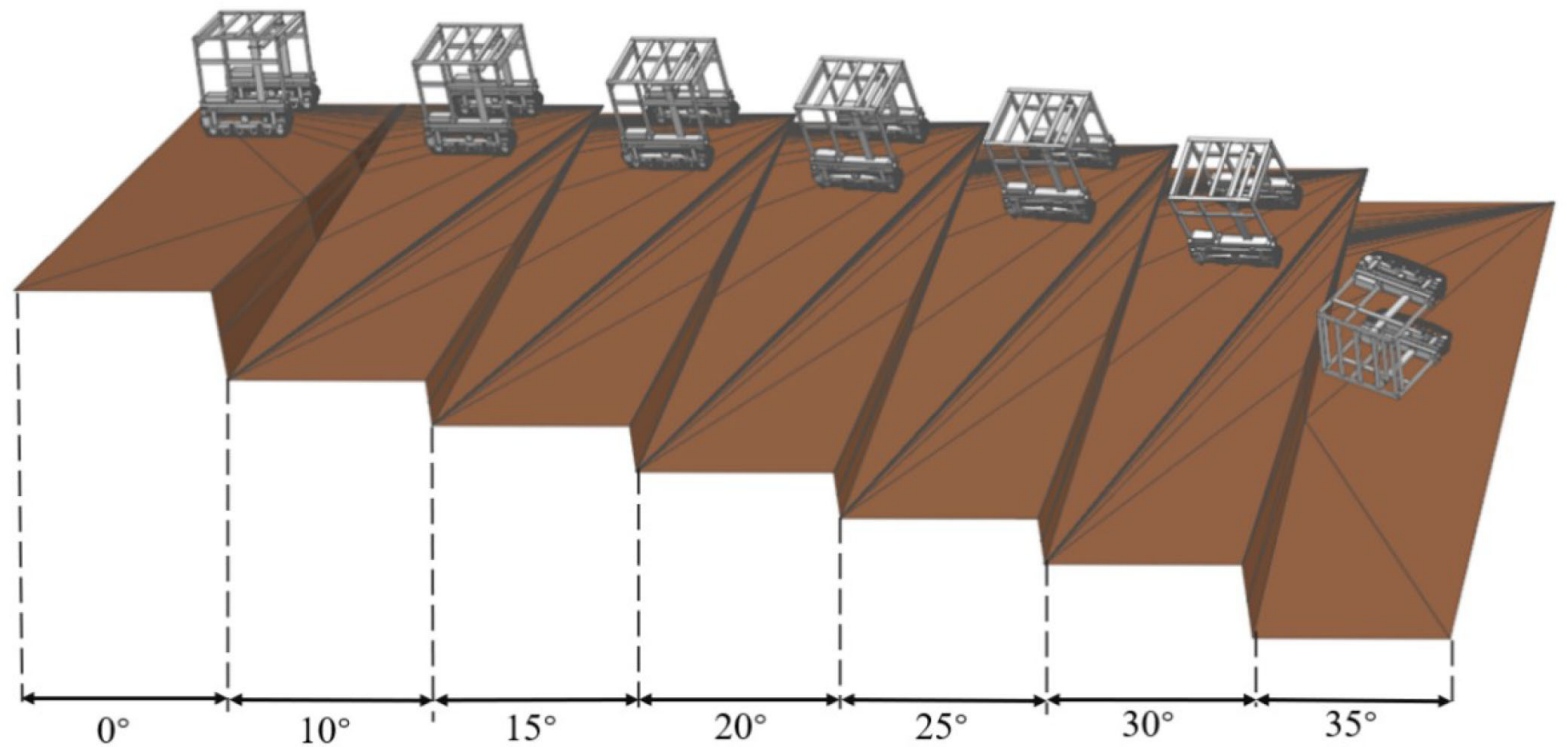
Simulation process of driving on continuous horizontal inclined road surface.

To evaluate the antirollover performance of the high-clearance crawler chassis on lateral slopes and account for the chassis slip behavior, terrain with a continuous lateral slope was modeled under sandy soil conditions (**Figure 13**). The lateral slope angle increased gradually from 0° to 35°. The test was performed at a crawler speed of 3.6 km/h. During the test, the track speeds were manually controlled to maintain the direction perpendicular to the slope. (**Figure 13**).

**Figure 14:**
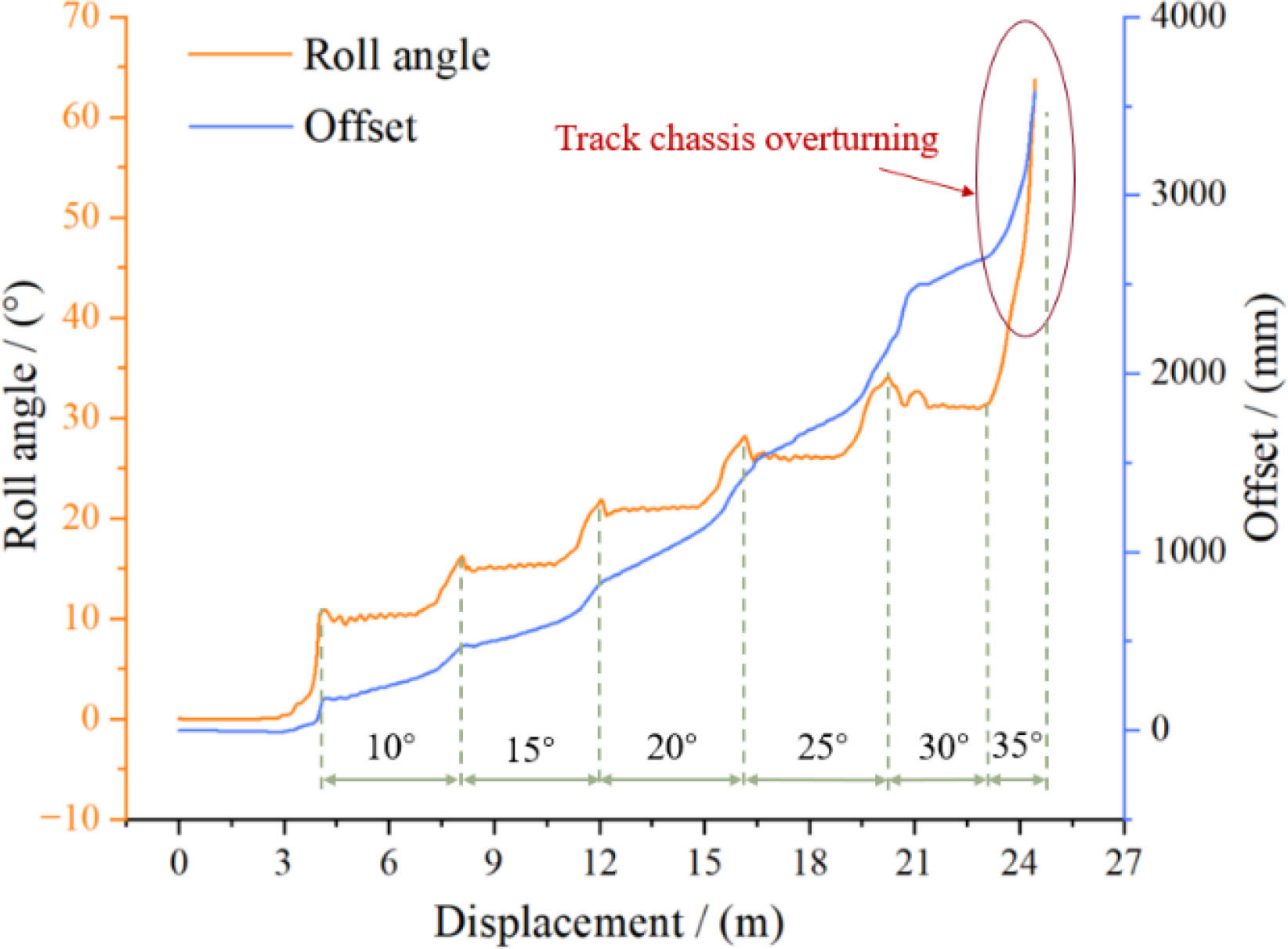
Simulation results of continuous lateral slope road driving.

The simulation results presented in **Figure 14** reveal that the roll angle of the crawler chassis remains relatively constant during operation on lateral slopes from 0° to 30°. However, rollover occurs when the slope transitions from 30° to 35°. These findings demonstrate that the chassis can operate safely on lateral slopes up to 30°, where it exhibits only manageable lateral slip and yaw that can be corrected through manual speed and directional adjustments. Beyond 30°, however, the risk of overturning increases significantly. (**Figure 14**).

#### 3.1.3 Obstacle-crossing stability analysis

**Figure 15:**
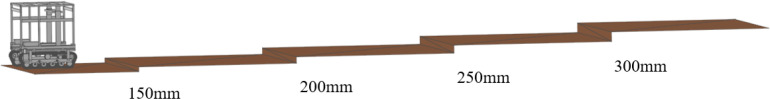
Obstacle-crossing condition road surface model.

To evaluate the driving stability of the crawler chassis under typical obstacle-crossing conditions in hilly and mountainous terrain and to examine the effect of the longitudinal center-of-gravity adjustment device on obstacle negotiation performance, vertical step models with heights of 150, 200, 250, and 300 mm were incrementally established on a sandy soil road for simulations (**Figure 15**). A distance between successive steps of 5 meters was maintained to ensure that the chassis could fully stabilize after it crossed one obstacle before the next was encountered. Additionally, a vehicle speed of 3.6 km/h was used to conduct comparative simulations with and without longitudinal center-of-gravity adjustment to assess the influence of the adjustment mechanism on obstacle-crossing stability. (**Figure 15**).

**Figure 16:**
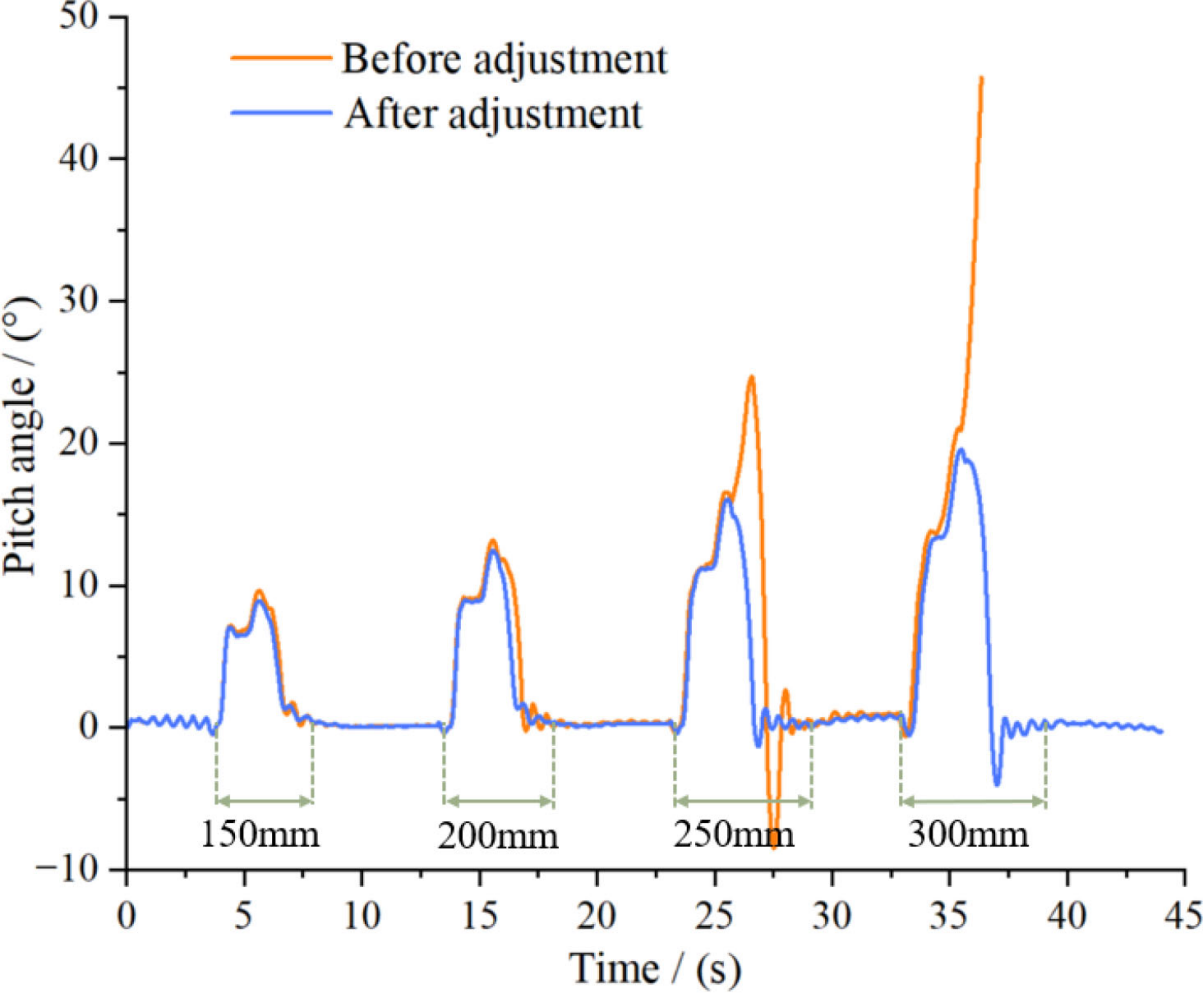
Pitch angle during obstacle clearance process.

As shown in the simulation results in **Figure 16**, when the longitudinal center-of-gravity adjustment device is inactive, the pitch angle amplitude of the crawler chassis increases with step height, and a corresponding increase in ground impact upon landing also occurs. When crossing a 250-mm step, a negative pitch angle occurs, indicating a tendency for the chassis to tilt forward after it contacts the ground. Although this angle remains relatively small and does not significantly hinder traversal, the chassis overturns when it negotiates a 300-mm step. After center-of-gravity adjustment, the variation in the pitch angle is reduced, allowing the crawler chassis to safely cross taller steps. However, the increased negative pitch angle after landing reflects a greater impact load. Therefore, reverse actuation of the center-of-gravity adjustment device should be fully utilized to counteract the increase in the negative pitch angle and landing stability. (**Figure 16**).

#### 3.1.4 Simulation analysis of trench-crossing performance

**Figure 17:**
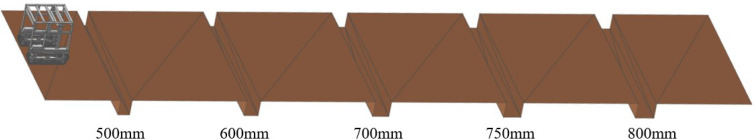
Cross-gully condition pavement model.

To evaluate the stability of the high-clearance crawler chassis under typical trench-crossing conditions in hilly and mountainous terrain, the influence of travel speed and longitudinal center-of-gravity position on trench-crossing performance was analyzed. Trench models with widths of 500, 600, 700, 750, and 800 mm were sequentially constructed on a sandy soil surface. To ensure that the chassis completed each crossing and stabilized before the next trench was encountered, a spacing of 5 meters was maintained between successive trenches (**Figure 17**). The chassis was tested at speeds of 1.8, 3.6, and 5.4 km/h to assess the effect of travel velocity on trench traversal performance. Additionally, a comparative simulation was conducted at 3.6 km/h with and without center-of-gravity adjustment to evaluate the influence of the center-of-gravity adjustment device on trench-crossing behavior. (**Figure 17**).

**Figure 18:**
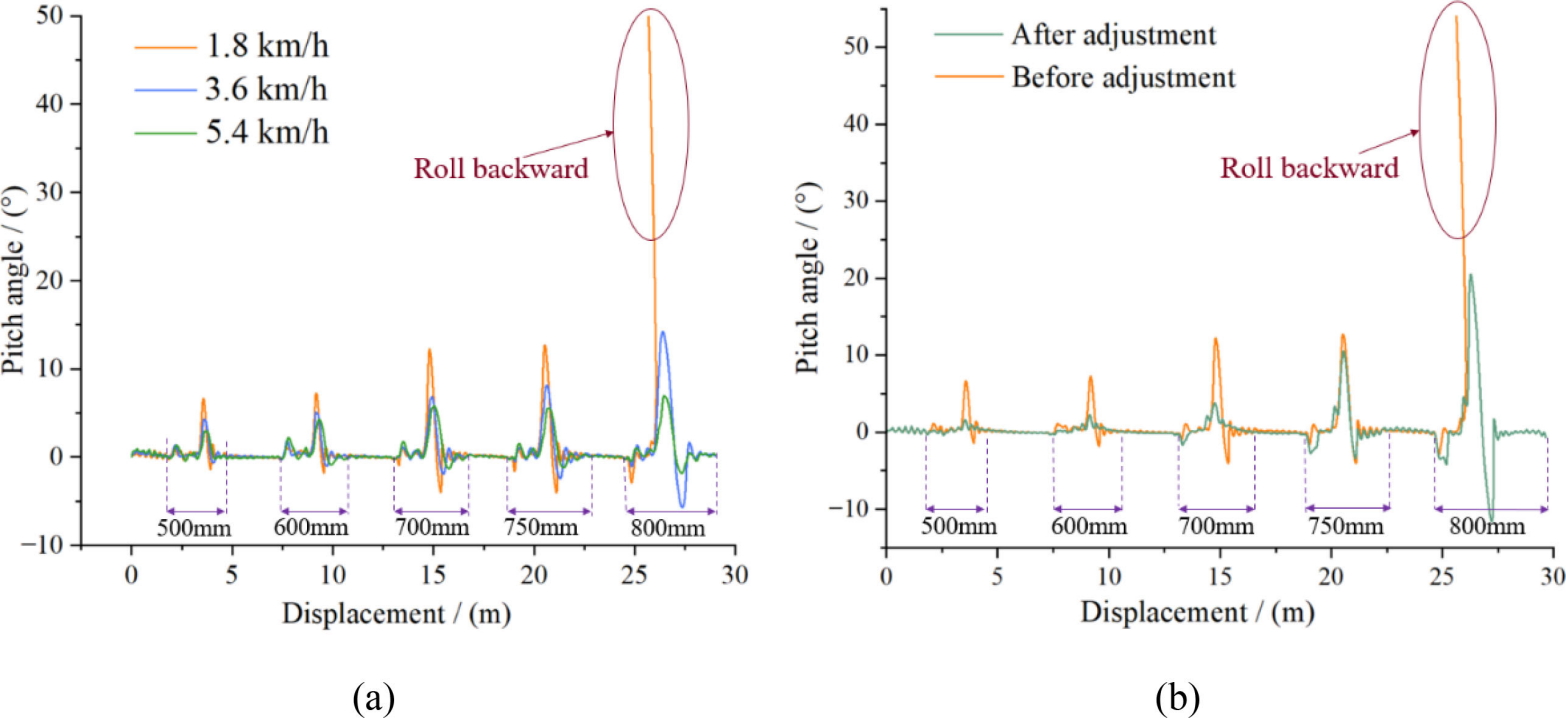
Simulation results of pitch angle during the crossing of the trench. **(a)** Simulation results at different speeds; **(b)** Simulation results before and after adjustment.

The simulation results presented in **Figure 18** demonstrate that as the trench width increases, the maximum pitch angle of the crawler chassis during the crossing process progressively increases, and the time required to regain stability after crossing is extended. When traversing trenches of identical width at higher speeds, the maximum pitch angle is reduced. This finding indicates that increased velocity facilitates quicker passage through the insufficient ground support phase, thereby enabling the chassis to cross wider trenches. The longitudinal center-of-gravity adjustment device effectively suppresses the pitch angle induced by the overturning moment during trench crossing, enhances antitipping capability, and permits the safe traversal of wider trenches. However, attention should also be given to the impact generated upon track–ground contact after crossing, as well as instability factors such as inertia-induced forward tipping.

Instability occurs during the trench-crossing process primarily in the fourth stage, which is characterized by the suspension of the driving wheels and minimal contact between the rear end of the tracks and the ground. This instability arises mainly because the drive motors and reducers are concentrated at the rear of the chassis, generating a substantial overturning moment that leads to a significant increase in the pitch angle. The longitudinal center-of-gravity adjustment device effectively counteracts this overturning moment during trench crossing, reduces the pitch angle, and increases the maximum negotiable trench width. (**Figure 18**).

### 3.2 Field tests

To validate the trafficability of the designed high-clearance electric-powered crawler chassis with posture adjustment functionality under complex terrain conditions in hilly and mountainous regions (Sun et al., 2020; Wang et al., 2022; Wang et al., 2024), obstacle negotiation tests and contour slope driving performance evaluations were conducted in accordance with the Chinese National Standard GB/T 3871-2004, “Test Methods for Agricultural Wheeled and Crawler Tractors.” Furthermore, the practical effectiveness of the longitudinal center-of-gravity adjustment device was experimentally verified. The test speed is determined based on the typical operation conditions in hilly and mountainous areas such as spraying pesticides and weeding, as well as the maximum driving speed designed for this chassis. The main instruments and measuring tools used in the field tests include a meter stick, a steel tape measure, a slope measuring instrument, a protractor, and a colored rope.

#### 3.2.1 Gradient climbing performance test

**Figure 19:**
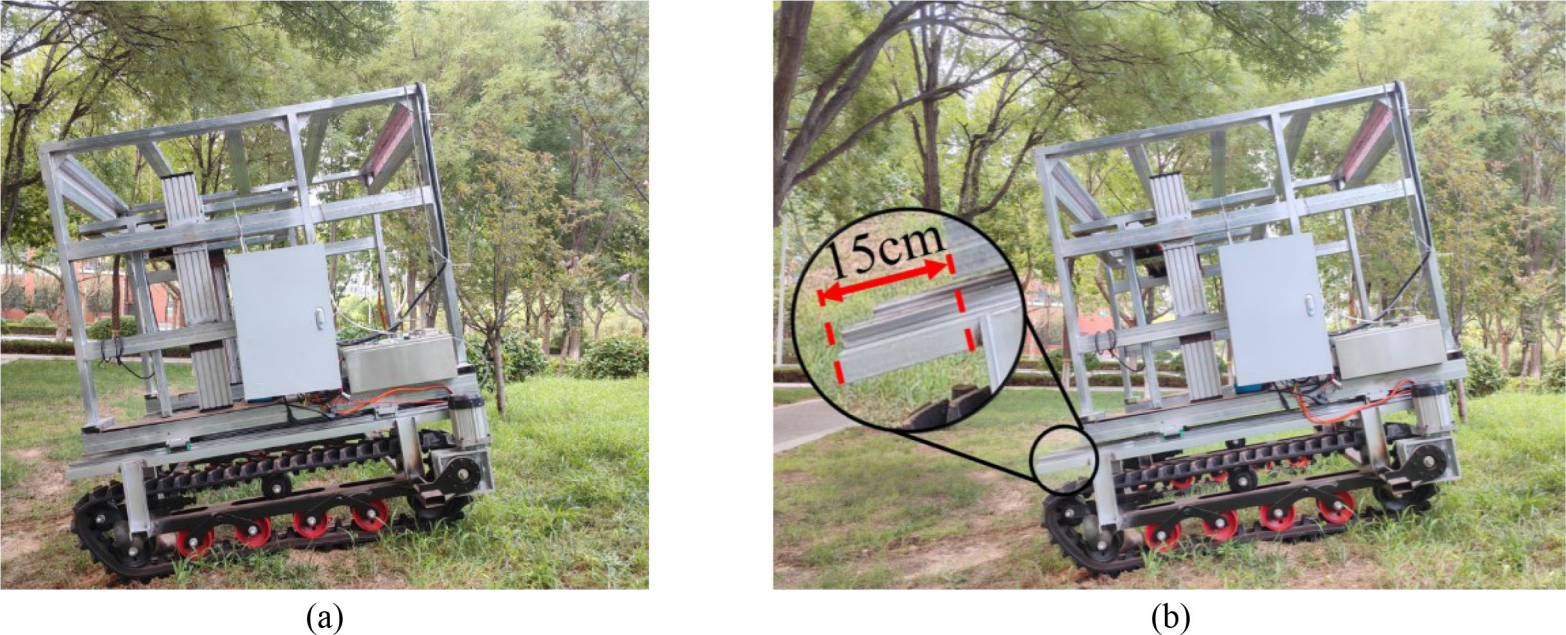
Field tests on climbing performance. **(a)** Before adjustment; **(b)** After adjustment.

To evaluate the driving performance of the chassis under longitudinal slope conditions and assess the improvement in climbing ability afforded by the longitudinal center-of-gravity adjustment device, a test slope with gradients ranging from 15° to 30° was selected. The crawler was maintained at a constant speed of 3.6 km/h during the tests. The lift-off tendency of the front support point was used as the criterion for determining the maximum climbable slope angle (**Figure 19**). Experiments were conducted with two configurations: with the longitudinal center-of-gravity adjustment device engaged and disengaged. Each configuration was tested three times. The maximum negotiable slopes were recorded, and average values were calculated (**Figure 19**; **Table 3**).

**Table 3:** Field test results of climbing performance.

Status of the longitudinal adjustment device for the center of gravity	Order number	Climbing slope/(°)	Success/ Failure	Maximum climbing gradient/(°)
The center of gravity remains unadjusted.	1	20	Success	30
	2	25	Success	
	3	30	Success	
	4	35	Failure	
The center of gravity remains adjusted.	1	20	Success	35
	2	25	Success	
	3	30	Success	
	4	35	Success	

When the center-of-gravity adjustment device was deactivated, the maximum slope angle at which the chassis could safely traverse was 30°. After the device was activated, the maximum safe slope angle increased to 35°, representing an improvement of 5°compared with that under the nonadjusted condition. These findings demonstrate that the longitudinal center-of-gravity adjustment device increases the antitipping moment by shifting the center of gravity forward, thereby effectively improving longitudinal slope-climbing stability. The experimental values were slightly lower than the simulation results, primarily because of the soft soil conditions at the test site and localized subsidence on the slope. These factors resulted in an uneven distribution of ground contact pressure under the tracks, reducing effective adhesion.

#### 3.2.2 Contour line slope driving performance test

**Figure 20:**
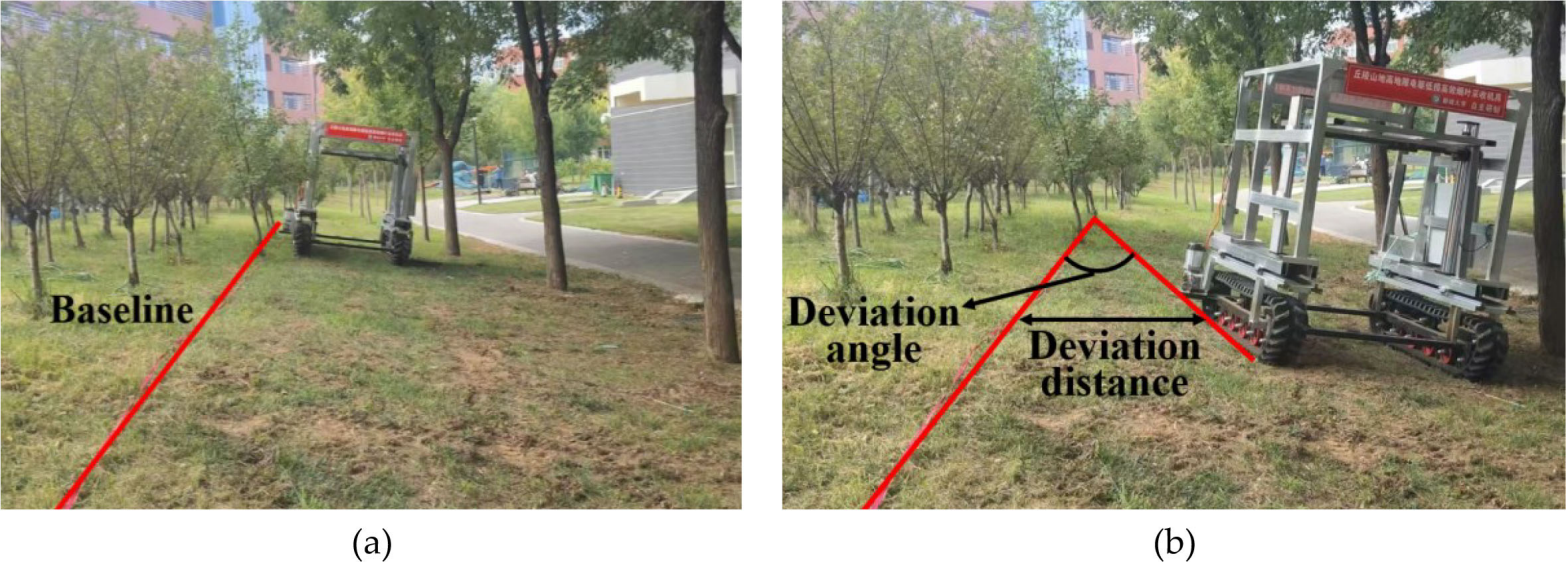
Contour driving test. **(a)** stage 1; **(b)** stage 2.

A test site with a contour slope gradient of 10° to 20° was selected, and a reference baseline was established on the uphill side. During testing, the prototype traveled at a constant speed of 3.6 km/h along the contour direction for a distance of 20 m. The lateral offset distance and deviation angle of the outer crawler relative to the baseline were measured at the endpoint. Each slope gradient was tested three times, and the average values were calculated (**Figure 20**; **Table 4**).

**Table 4:** Field test results of contour line slope driving.

Slope (°)	Order number	Offset (mm)	Average offset/(mm)	Deviation angle/(°)	Average deviation angle/(°)
10	1	1030	1018.3	10	9.3
	2	955		9	
	3	1070		9	
15	1	2175	2166.7	22	20.3
	2	2230		20	
	3	2095		19	
20	1	3060	3150	33	34.6
	2	3220		37	
	3	3170		34	

As the slope gradient increases, both the lateral offset distance and the yaw rate increase markedly. The measured yaw rates were 9.3° on a 10° slope, 20.3° on a 15° slope, and 34.6° on a 20° slope. This trend is attributed primarily to the increased lateral component of gravity on steeper slopes, which disrupts the adhesion balance between the two tracks and exacerbates skidding and deviation. Furthermore, the experimental results suggest that the rate of lateral offset and the yaw angle are influenced to some extent by ground unevenness.

#### 3.2.3 Obstacle-crossing performance test

**Figure 21:**
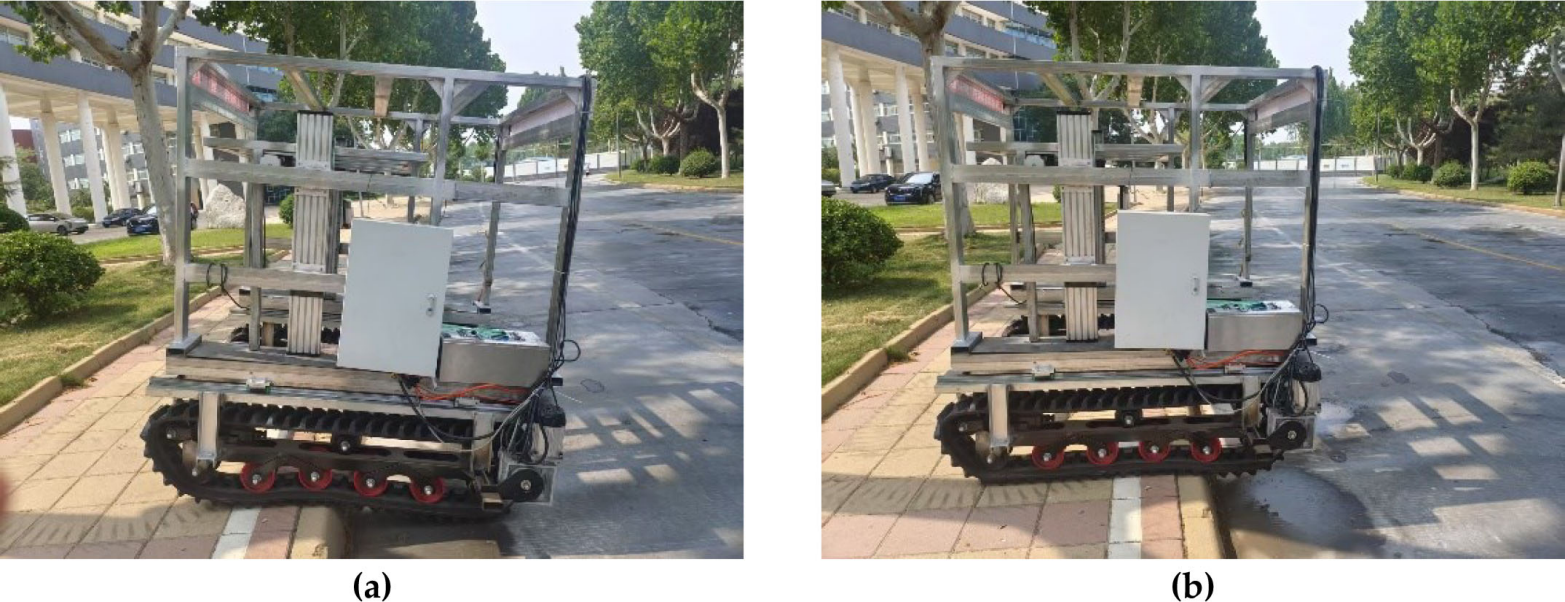
Obstacle-crossing performance test. **(a)** stage 1; **(b)** stage 2.

In the field test, vertical steps of varying heights were selected to evaluate the obstacle-crossing performance. The vehicle approached the steps at a constant speed of 3.6 km/h, and the step height was incrementally increased. Tests were conducted using two configurations: with the longitudinal center-of-gravity adjustment device enabled and disabled. The maximum safe obstacle-crossing height was recorded for each condition (**Figure 21**; **Table 5**).

**Table 5:** Field test results of obstacle-crossing performance.

Status of the longitudinal adjustment device for the center of gravity	Order number	Obstacle - crossing height/mm	Success/ Failure	Maximum obstacle - crossing height/mm
The center of gravity remains unadjusted	1	150	Success	250
	2	200	Success	
	3	250	Success	
	4	300	Failure	
The center of gravity remains adjusted	1	150	Success	300
	2	200	Success	
	3	250	Success	
	4	300	Success	

When the longitudinal center-of-gravity adjustment device was deactivated, the maximum obstacle height successfully traversed was 250 mm. With the device activated, the maximum surmountable obstacle height increased to 300 mm—an improvement of 50 mm. This finding demonstrates that longitudinal adjustment of the center of gravity enhances the anti-overturning moment and reduces pitch angle fluctuations during step descent. The field test results were lower than the simulation values, primarily because the test steps were constructed from rigid, nondeformable pavement material, which generated greater impact forces during crossing.

#### 3.2.4 Trench-crossing performance test

**Figure 22:**
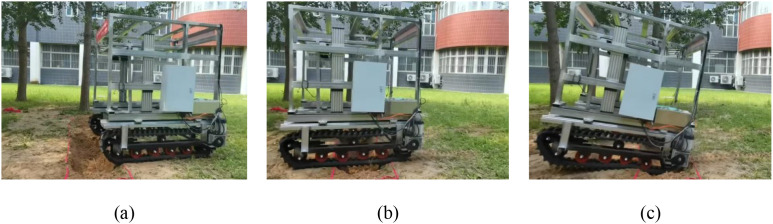
Field tests on trench-crossing performance. **(a)** stage 1; **(b)** stage 2; **(c)** stage 3.

Rectangular trenches were constructed on a level road surface to evaluate the trench-crossing performance. Testing began with relatively narrow trenches, and the width was gradually increased until the crawler chassis could no longer traverse the trench safely. Experiments were conducted using four configurations: 3.6 km/h (the center of gravity remains unadjusted), 3.6 km/h (the center of gravity remains adjusted), 5.4 km/h (the center of gravity remains unadjusted), 5.4 km/h (the center of gravity remains adjusted). The maximum width at which the trench could be crossed safely was recorded for each configuration (**Figure 22**; **Table 6**).

**Table 6:** Field test results of trench-crossing performance.

Trench - crossing speed (km/h)	Order number	Cross - trench width/mm	Success/ Failure	Maximum cross-trench width/mm
3.6 (The center of gravity remains unadjusted)	1	400	Success	600
	2	500	Success	
	3	600	Success	
	4	700	Failure	
3.6 (The center of gravity remains adjusted)	1	400	Success	700
	2	500	Success	
	3	600	Success	
	4	700	Success	
5.4 (The center of gravity remains unadjusted)	1	500	Success	700
	2	600	Success	
	3	700	Success	
	4	750	Failure	
5.4 (The center of gravity remains adjusted)	1	500	Success	750
	2	600	Success	
	3	700	Success	
	4	750	Success	

While operating at 3.6 km/h, the maximum trench width that could be crossed without activating the longitudinal center-of-gravity adjustment device was 600 mm. After activation, the maximum width increased to 700 mm, an improvement of 100 mm, indicating that shifting the center of gravity backward effectively suppressed the forward pitching moment during trench traversal. At a speed of 5.4 km/h, the maximum trench width that could be crossed without center-of-gravity adjustment reached 700 mm, which is marginally greater than that achieved at lower speeds. This improvement can be attributed to the reduced suspension time of the tracks and diminished pitch angle fluctuations at higher speeds. When the longitudinal center-of-gravity adjustment device was activated during operation at 5.4 km/h, the maximum negotiable trench width increased further to 750 mm, demonstrating that the device also enhances trench-crossing performance at elevated travel speeds. Notably, discrepancies were observed between the field test results and simulation values. These discrepancies are due primarily to local collapse of the soft soil at the edges of the trench, which compromised support stability during actual crossing attempts.

## 4 Conclusion

(1) In this study, a high-clearance electric-driven crawler chassis featuring a longitudinally adjustable center of gravity was developed. The gantry-type frame structure provides a ground clearance of 1550 mm, meeting the requirements for interrow operations in tall-stem crops grown in hilly and mountainous areas, as well as in orchards and tea plantations. A real-time slope detection system incorporating an inclination sensor and an STM32 microcontroller controls an electric push rod that longitudinally displaces the upper frame by ±350 mm relative to the crawler traveling system. This design enables active adjustment of the center of gravity, which increases longitudinal stability during slope climbing, obstacle crossing, and trench traversal over complex terrain. Additionally, an implement pose adjustment mechanism using dual sliding tables for vertical lift and a single sliding table for horizontal displacement was designed. This system allows rapid mounting and the interchange of implements to accommodate diverse agronomic needs while ensuring precise vertical and horizontal positioning, thereby increasing the accuracy and efficiency of interrow operations.

(2) Static and kinematic analyses were performed on the high-clearance electric-driven crawler chassis, and mechanical models were established for typical working conditions, including longitudinal slope climbing, contour slope traversal, vertical step crossing, and trench negotiation. The influence of structural parameters on the driving stability and trafficability of the chassis was thoroughly investigated. The feasibility of the longitudinal center-of-gravity adjustment device was validated through theoretical modeling. By adjusting the longitudinal position of the center of gravity, the device effectively increases the ultimate pitch angle, suppresses the risk of backward tipping during ascent and forward tipping during descent, reduces pitch fluctuation during trench crossing, and improves overall trench-crossing stability.

(3) A virtual prototype of the chassis was developed using the multibody dynamics software RecurDyn. Dynamic simulations were performed with two typical terrain models—sandy soil and clay—to evaluate its performance in characteristic scenarios such as slope climbing, contour slope traversal, obstacle-crossing, and obstacle negotiation. The effect of the longitudinal center-of-gravity adjustment device on improving the mobility and stability of the crawler chassis was assessed by monitoring dynamic parameters, including the velocity trajectory of the center of mass, pitch angle, roll angle, and center-of-mass displacement. The multibody dynamics simulation results were in good agreement with the theoretical analysis results.

(4) Field tests were carried out to validate the theoretical analysis and simulation results. Experiments performed under typical working conditions—such as slope climbing, contour driving, obstacle crossing, and trench-crossing—demonstrated that engaging the longitudinal center-of-gravity adjustment device increased the maximum safe slope-climbing angle from 30° to 35°, the maximum trench-crossing width at 3.6 km/h from 600 to 700 mm, and the maximum surmountable vertical step height from 250 to 300 mm. Furthermore, at 5.4 km/h, the maximum trench-crossing width on the lateral slopes increased from 700 to 750 mm. During lateral slope travel, slip and deviation phenomena were observed, the extent of which was influenced by both the gradient and the ground surface roughness. Although some discrepancies were noted between the experimental and simulation results—primarily because of differences between actual field conditions (e.g., soil softness and localized collapse) and the idealized parameters used in simulations—the field outcomes consistently validated the rationality of the theoretical model, the reliability of the simulation predictions, and the practical effectiveness of the chassis design and its longitudinal center-of-gravity adjustment device.

## Data Availability

The original contributions presented in the study are included in the article/supplementary material. Further inquiries can be directed to the corresponding author.

## References

[B1] Calciolari L. Pantano M. Pantano G. Concheri G. (2024). “ Preliminary design and analysis of a modular autonomous mobile robot for vineyard operations,” in International Conference on Safety, Health and Welfare in Agriculture and Agro-food Systems. (Cham: Springer Nature Switzerland). 285–295.

[B2] Chen Y. Mao E. Li W. Zhang S. Song Z. Yang S. . (2020). Design and experiment of a high-clearance self-propelled sprayer chassis. Int. J. Agric. Biol. Eng. 13, 71–80. doi: 10.25165/j.ijabe.20201302.5262

[B3] Chen Y. Wang Z. Zhang H. Liu X. Li H. Sun W. . (2024). Investigation of the traveling performance of the tracked chassis of a potato combine harvester in hilly and mountainous areas. Agriculture 14, 1625. doi: 10.3390/agriculture14091625, PMID: 41725453

[B4] Coneo R. J. C. de Araujo W. L. Garcia A. P. dos Santos A. A. (2025). Rubber-tracked crawler undercarriage multibody dynamic simulation for agriculture operation. Sci. Rep. 15, 24829. doi: 10.1038/s41598-025-08213-w, PMID: 40640207 PMC12246062

[B5] Cui Z. Guan C. Chen Y. Gao Q. Yang Y. (2019). Design of small multi-functional electric crawler platform for greenhouse. Trans. Chin. Soc. Agric. Eng. (Trans. CSAE) 35, 48–57.

[B6] Ding R. Qi X. Chen X. Mei Y. Li A. (2025). The current development status of agricultural machinery chassis in hilly and mountainous regions. Appl. Sci. 15, 7505. doi: 10.3390/app15137505, PMID: 41725453

[B7] Gao Q. Pan D. Zhang X. Deng F. Huang D. Wang L. (2020). Design and simulation of entire track modular unmanned agricultural power chassis. Trans. Chin. Soc. For. Agric. Mach. 51, 561–570.

[B8] Han Z. Zhu L. Yuan Y. Zhao B. Fang X. Wang D. (2022a). Design and test of transport vehicle for hillside orchards based on center of gravity regulation. Trans. Chin. Soc. For. Agric. Mach. 53, 430–442.

[B9] Han Z. Zhu L. Yuan Y. Zhao B. Fang C. Zhang T. (2022b). Analysis of slope trafficability and optimized design of crawler chassis in hillside orchard. Trans. Chin. Soc. For. Agric. Mach. 53, 413–421+448.

[B10] He S. Shen Y. Zhang Y. Liu H. (2023). Development and evaluation of 4WSS electric-driven chassis for high-clearance sprayer. Front. Plant Sci. 14. doi: 10.3389/fpls.2023.1258744, PMID: 37841626 PMC10568742

[B11] Jiang Y. Wang R. Ding R. Sun Z. Jiang Y. Liu W. (2025). Research review of agricultural machinery power chassis in hilly and mountainous areas. Agriculture 15, 1158. doi: 10.3390/agriculture15111158, PMID: 41725453

[B12] Jin M. Zhang M. Wang G. Liang S. Wu C. He R. (2022). Analysis and simulation of wheel-track high clearance chassis of rape windrower. Agriculture 12, 1150. doi: 10.3390/agriculture12081150, PMID: 41725453

[B13] Li H. Chen L. Zhang Z. (2022). A study on the utilization rate and influencing factors of small agricultural machinery: Evidence from 10 hilly and mountainous Provinces in China. Agriculture 13, 51. doi: 10.3390/agriculture13010051, PMID: 41725453

[B14] Li C. Xiang S. Ye K. Luo X. Zhu C. Li J. . (2024). Design and experiment of an independent leg-type chassis vehicle attitude adjustment system. Agriculture 14, 1548. doi: 10.3390/agriculture14091548, PMID: 41725453

[B15] Li X. Yang F. Sun R. Peng Z. Shen X. Xu G. (2024). Design of a gantry crawler multifunctional operation platform for wine grape cultivation. Agriculture 14, 1587. doi: 10.3390/agriculture14091587, PMID: 41725453

[B16] Lin X. Zhu Y. Xie Z. (2022). Mechanics modeling and simulation analysis of a novel articulated chassis for forestry. Sustainability 14, 16118. doi: 10.3390/su142316118, PMID: 41725453

[B17] Mengoli D. Tazzari R. Marconi L. (2020). “ Autonomous robotic platform for precision orchard management: architecture and software perspective,” in 2020 IEEE International Workshop on Metrology for Agriculture and Forestry (MetroAgriFor). ( IEEE). 303–308.

[B18] Mou X. Luo Q. Ma G. Wan F. He C. Yue Y. . (2023). Simulation analysis and testing of tracked universal chassis traversability in hilly mountainous orchards. Agriculture 13, 1458. doi: 10.3390/agriculture13071458, PMID: 41725453

[B19] Pan K. Zhang Q. Wang Z. Wang S. Zhou A. You Y. . (2024). Method for the posture control of bionic mechanical wheel-legged vehicles in hilly and mountainous areas. Int. J. Agric. Biol. Eng. 17, 151–162. doi: 10.25165/j.ijabe.20241705.8383

[B20] Paul A. Machavaram R. (2024). Design of an adjustable chassis for a track type combine harvester. Cogent. Eng. 11, 2353811. doi: 10.1080/23311916.2024.2353811, PMID: 41909888

[B21] Peng Y. X. Liao K. Xu S. Y. Chen F. Li L. J. Tang G. C. . (2024). Development of a wheeled integrated machine for *Camellia oleifera* fruit harvesting in hilly and mountainous areas. Trans. Chin. Soc. Agric. Eng. (Trans.CSAE). 40:31–38. doi: 10.11975/j.issn.1002-6819.202404037

[B22] Roul A. K. Singh D. (2022). Development and stability analysis of a self-propelled high clearance multi-utility vehicle. J. Agric. Eng. (India) 59, 18–30. doi: 10.52151/jae2022591.1762

[B23] Su C. Huang H. (2024). Enhancing terrain adaptability of micro tracked chassis: A structural design and performance evaluation. Mechanics 30, 544–559. doi: 10.5755/j02.mech.36883

[B24] Sun J. Chu G. Pan G. Meng C. Liu Z. Yang F. (2021). Design and performance test of remote control omnidirectional leveling hillside crawler tractor. Trans. Chin. Soc. For. Agric. Mach. 52, 358–369.

[B25] Sun J. Liu Z. Yang F. Sun Q. Liu Q. Luo P. (2023). Research review of agricultural equipment and slope operation key technologies in hilly and mountains region. Trans. Chin. Soc. For. Agric. Mach. 54, 1–18.

[B26] Sun J. Meng C. Zhang Y. Chu G. Zhang Y. Yang F. . (2020). Design and physical model experiment of an attitude adjustment device for a crawler tractor in hilly and mountainous regions. Inf. Process. Agric. 7, 466–478. doi: 10.1016/j.inpa.2020.02.004, PMID: 41940325

[B27] Sun S. Wu J. Ren C. Tang H. Chu J. (2020). Chassis trafficability simulation and experiment of a ly1352jp forest tracked vehicle. J. For. Res. 32, 1315–1325 doi: 10.1007/s11676-019-01095-5, PMID: 41940407

[B28] Wang Y. Lin J. Zhang L. Wang T. Xu H. Qi Y. (2022). Stable obstacle avoidance strategy for crawler-type intelligent transportation vehicle in non-structural environment based on attention-learning. IEEE Trans. Intell. Transp. Syst. 24, 7813–7830. doi: 10.1109/tits.2022.3226493, PMID: 41116384

[B29] Wang Y. Liu H. Song L. Liang L. Zhang Y. Yu Z. . (2024). Analysis of the driving stability of a multi-model driven electric-tracked intelligent detection device. Actuators 13, 527. doi: 10.3390/act13120527, PMID: 41725453

[B30] Wang W. Luo C. Zhang G. Fu J. Dong Z. Ji C. . (2022). Design and experiment of automatic navigation system for rice harvester with dual-motor crawler chassis. J. Huazhong. Agric. Univ. 41, 199–207.

[B31] Wang D. Zhao Y. You Y. Zhang X. Wang T. (2023). Design and experiment of self-propelled tracked chassis of king grass harvester for gentle sloping fields. Trans. Chin. Soc. For. Agric. Mach. 54, 178–187. doi: 10.13031/aim.202000405

[B32] Wu Z. Chen L. Wang Y. Luo K. Lai X. Zhang X. . (2025). Design and experiment on traversibility of traveling chassis of crawler self-propelled tea picker. Trans. Chin. Soc. For. Agric. Mach. 56, 474–483.

[B33] Xiang W. Tang J. Zhou J. Kou X. Fan Z. (2025). Design and test of power chassis of camellia oleifera harvester in hilly Area. J. Northwest. For. Univ. 40, 233–239+250.

[B34] Xie X. Han X. Zhang Z. Qin Y. Li Y. Yan Z. (2023). Structural design and test of arch waist dynamic chassis for hilly and mountainous areas. Int. J. Adv. Manuf. Technol. 127, 1921–1933. doi: 10.1007/s00170-022-10224-0, PMID: 41940407

[B35] Xu H. Chen Y. Hu M. Yu G. Zheng C. Zhang Z. . (2024). Design and test on high-gap wheeled agricultural chassis for harvesting broccoli. Eng. Res. Express. 6, 045104. doi: 10.1088/2631-8695/ad8537

[B36] Zhang Z. Deng Y. Wang F. Cao Q. Xie J. (2023). Trafficability analysis and scaling model experiment of self-propelled panax notoginseng combine harvester chassis. Trans. Chin. Soc. For. Agric. Mach. 54, 164–177. doi: 10.13031/aim.202000405

[B37] Zhang Z. Wang Y. Wen B. Guo S. Xie K. Wang C. (2024). Design and trafficability experiment of self-propelled panax notoginseng combine harvester. Trans. Chin. Soc. For. Agric. Mach. 55, 306–319. doi: 10.13031/aim.202000405

[B38] Zheng Y. Jiang S. Chen B. Lu H. Wan C. Kang F. (2020). Review on technology and equipment of mechanization in hilly orchard. Trans. Chin. Soc. For. Agric. Mach. 51, 1–20.

